# Spectral Slope and Lempel–Ziv Complexity as Robust Markers of Brain States during Sleep and Wakefulness

**DOI:** 10.1523/ENEURO.0259-23.2024

**Published:** 2024-03-25

**Authors:** Christopher Höhn, Michael A. Hahn, Janna D. Lendner, Kerstin Hoedlmoser

**Affiliations:** ^1^Laboratory for Sleep, Cognition and Consciousness Research, Department of Psychology, University of Salzburg, 5020 Salzburg, Austria; ^2^Centre for Cognitive Neuroscience Salzburg (CCNS), University of Salzburg, 5020 Salzburg, Austria; ^3^Hertie-Institute for Clinical Brain Research, University Medical Center Tübingen, 72076 Tübingen, Germany; ^4^Department of Anesthesiology and Intensive Care Medicine, University Medical Center Tübingen, 72076 Tübingen, Germany

**Keywords:** alertness, cognitive tasks, EEG, Lempel–Ziv complexity, sleep, spectral slope

## Abstract

Nonoscillatory measures of brain activity such as the spectral slope and Lempel–Ziv complexity are affected by many neurological disorders and modulated by sleep. A multitude of frequency ranges, particularly a broadband (encompassing the full spectrum) and a narrowband approach, have been used especially for estimating the spectral slope. However, the effects of choosing different frequency ranges have not yet been explored in detail. Here, we evaluated the impact of sleep stage and task engagement (resting, attention, and memory) on slope and complexity in a narrowband (30–45 Hz) and broadband (1–45 Hz) frequency range in 28 healthy male human subjects (21.54 ± 1.90 years) using a within-subject design over 2 weeks with three recording nights and days per subject. We strived to determine how different brain states and frequency ranges affect slope and complexity and how the two measures perform in comparison. In the broadband range, the slope steepened, and complexity decreased continuously from wakefulness to N3 sleep. REM sleep, however, was best discriminated by the narrowband slope. Importantly, slope and complexity also differed between tasks during wakefulness. While narrowband complexity decreased with task engagement, the slope flattened in both frequency ranges. Interestingly, only the narrowband slope was positively correlated with task performance. Our results show that slope and complexity are sensitive indices of brain state variations during wakefulness and sleep. However, the spectral slope yields more information and could be used for a greater variety of research questions than Lempel–Ziv complexity, especially when a narrowband frequency range is used.

## Significance Statement

We demonstrate that the spectral slope and Lempel–Ziv complexity differentiate between sleep stages, quiet wakefulness and active tasks, thus making them reliable noninvasive biomarkers of brain states. Critically, these markers were previously assessed in isolation only. Here, we provide evidence that they track highly similar information about the underlying brain state in a broad frequency range (1–45 Hz). Within this range, slope and complexity distinguish brain states better than in a more narrowband range (30–45 Hz). However, the slope calculated from the narrowband range is superior in differentiating REM from wakefulness and tracking behavioral performance. Our results demonstrate that the choice of frequency range critically affects the information reflected by the spectral slope and Lempel–Ziv complexity.

## Introduction

Neural oscillations are a dominant electrophysiological signature of human brain activity. For instance, quiet wakefulness is characterized by pronounced alpha-band activity (Klimesch et al., 1993; Jensen and Mazaheri, 2010), and sleep stages are defined by oscillatory events like sleep spindles and slow oscillations (Terzano et al., 2002; Richard et al., 2012). However, recent evidence suggests that non-oscillatory, irregular brain activity, assessed by Lempel–Ziv complexity (Lempel and Ziv, 1976; Welch, 1984) or the spectral slope (He, 2014), also carries meaningful information about electrophysiological variations across brain states.

The spectral slope is obtained in the frequency domain and reflects the steepness of the power spectrum. In contrast, Lempel–Ziv complexity (Welch, 1984) is computed in the time domain and reflects the regularity of a signal (Lau et al., 2022). Thus, Lempel–Ziv complexity is still strongly influenced by oscillatory activity (Extended Data Fig. 1-4), whereas the spectral slope captures mainly aperiodic activity (Donoghue et al., 2020).

Computational modeling has demonstrated that the spectral slope constitutes a marker of the brain's excitation to inhibition (E/I) balance (Gao et al., 2017), which is impaired in a variety of clinical conditions (Rubenstein and Merzenich, 2003; Gao and Penzes, 2015; Robertson et al., 2019; Karalunas et al., 2022). Interestingly, some disorders, for instance, epilepsy, have been associated not only with alterations in the spectral slope but also in complexity (Wong, 2010; Aarabi and He, 2012; Zhu et al., 2017). Overall, previous studies suggest that both, spectral slope and Lempel–Ziv complexity, capture brain state changes in similar ways. The slope steepens (i.e., decreases) under anesthesia (Gao et al., 2017; Colombo et al., 2019; Lendner et al., 2020; Waschke et al., 2021) and during sleep (Ma et al., 2018; Miskovic et al., 2019; Lendner et al., 2020). Similarly, Lempel–Ziv complexity is reduced under anesthesia (Zhang et al., 2001; Ferenets et al., 2007) and with increasing sleep depth (Andrillon et al., 2016; Schartner et al., 2017). Evidence from Waschke et al. (2021) suggests that the spectral slope further tracks the level of attention, whereby faster response times are indexed by flatter slopes. This is in line with work demonstrating that the slope is indicative of cognitive processing speed (Ouyang et al., 2020; Pathania et al., 2022) and modulated by cognitive decline (Voytek et al., 2015; Voytek and Knight, 2015; Dave et al., 2018). Relatedly, higher task-related Lempel–Ziv complexity has also been suggested to track higher processing speed (Mediano et al., 2021).

A big issue, however, that has hampered the assessment of and comparability between slope and complexity is the huge heterogeneity in frequency ranges used to calculate these measures. While there will be no frequency range without potential confounds, it is important to compare different frequency ranges and calculation settings to gain a better understanding of how the choice of a certain frequency range affects the data. Particularly for the spectral slope, researchers have argued either in favor of broadband (Podvalny et al., 2015; Waschke et al., 2021; Karalunas et al., 2022) or more narrowband (Gao et al., 2017; Lendner et al., 2020) frequency ranges, commonly within 1–45/50 Hz. While broadband ranges (e.g., 1–45 Hz) encompass more of the total signal and result in better overall fits (Donoghue et al., 2020; Gerster et al., 2022), narrowband ranges (e.g., 30–45 Hz) are less affected by slower oscillatory activity and reflect mostly aperiodic activity (Gao et al., 2017; Lendner et al., 2020). But also regarding complexity, recent evidence demonstrated that this metric is strongly affected by different frequency contents and might be mainly driven by lower frequencies (González et al., 2022).

Taken together, a functional overlap between the spectral slope and Lempel–Ziv complexity is suggested in the literature. However, direct comparisons between the two measures are rare and limited to quiet wakefulness and anesthesia (Medel et al., 2023). Thus, even though slope and complexity are both biomarkers of arousal that are similarly modulated by changes in brain states, it is yet unclear how they behave in comparison. Moreover, it remains unclear how the selection of different frequency ranges affects the information captured by them.

Here, we leverage a within-subject design with multiple sleep and wake recordings over 14 days (1) to investigate whether spectral slope and Lempel–Ziv complexity are modulated by sleep stages and tasks during wakefulness and (2) to assess their significance for cognition in different frequency ranges. Additionally, we aim (3) to evaluate which parameter might be better suited under different circumstances and for different research questions. Finally, by using multiple recordings per subject, we assess the stability of these measures as indices of underlying brain states.

## Materials and Methods

### Participants and inclusion criteria

We recorded data from 28 biologically male participants (18–25 years; mean age, 21.54 ± 1.90 years) to avoid previously reported sex effects on sleep, attributed to hormonal variations (Alonso et al., 2021; Plamberger et al., 2021). Final sample sizes varied for each analysis between *N* = 26–28 as some participants had missing data for specific tasks or timepoints (the exact sample size for each analysis is provided in the corresponding figure caption). All participants were free of medication and did not suffer from a mental or physiological illness or reported sleep problems. They adhered to a regular sleep–wake rhythm (i.e., regular bedtimes with ∼8 h of sleep per night) and refrained from drug use and above-average caffeine consumption (more than three cups of coffee per day) during participation. For screening purposes, all subjects filled in an entrance questionnaire in which we checked for sleep quality, mood, anxiety, perceived stress level, and chronotype to exclude individuals with clinical symptoms in advance (compare Extended Data Fig. 1-1). Written informed consent was obtained from every participant, and they were remunerated with either 100€ and 16 h course credit or 50€ and 24 h course credit. The study was approved by the local ethics committee and conducted in agreement with the Declaration of Helsinki.

### Experimental protocol

#### Study design

**Figure 1.**
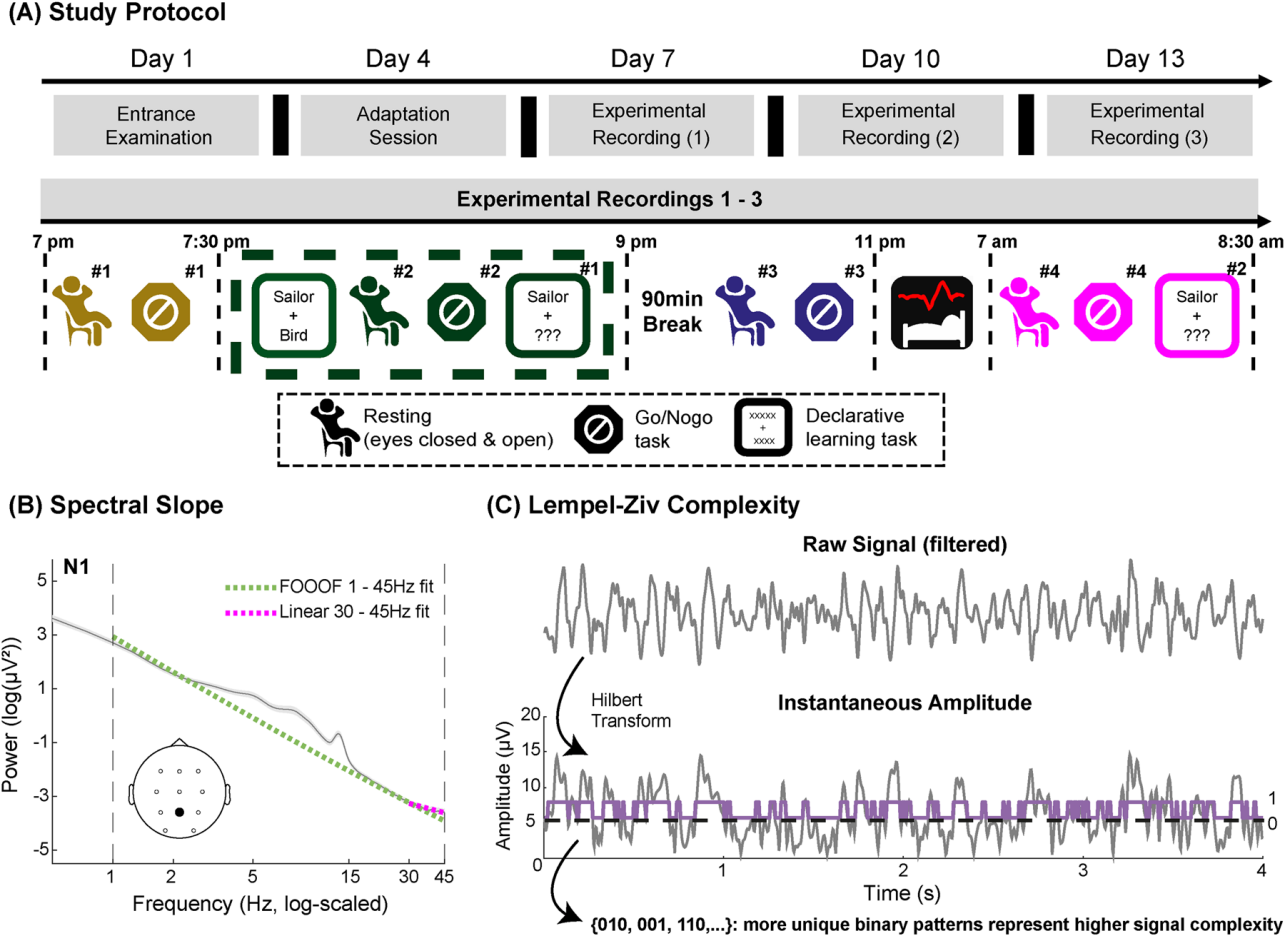
***A***, Overview of the experimental protocol. EEG was recorded throughout all tasks and during sleep (with full-night polysomnography) on the experimental days 7, 10, and 13 (see Extended Data Fig. 1-2 for the general sleep architecture on the different nights). The tasks, which are highlighted by a dashed, dark-green rectangle were primarily used to analyze the effects of engagement in different cognitive tasks during wakefulness. The adaptation night only served familiarization purposes and was not included in any of the analyses. An overview of the results from the screening entrance questionnaire is presented in Extended Data Figure 1-1. ***B***, Example of the spectral slope estimation during N1 sleep. For illustration purposes, data are shown for the electrode Pz averaged over all subjects and sleep recordings. The spectral slope was fitted within 1–45 Hz (broadband, dashed green line) and 30–45 Hz (narrowband, dashed pink line). ***C***, Schematic overview of the Lempel–Ziv complexity calculation based on a random 4 s epoch from electrode Pz of a subject during resting with closed eyes. First, the raw signal, filtered within a certain frequency range, is Hilbert transformed. Second, the resulting data is binarized around its median amplitude and stored as a vector of zeros and ones. Lastly, the Lempel–Ziv–Welch algorithm (Welch, 1984) is applied on this binary sequence in order to obtain a complexity value, which is driven by the number of unique repetitions of ones and zeros. The effect of signal regularity on Lempel–Ziv complexity and the spectral slope is further demonstrated in Extended Data Figure 1-4. For an overview of the number of epochs that were used for all analyses, see Extended Data Figure 1-3.

Each subject participated over a time span of 14 days (for an outline of the protocol, see Fig. 1). From that day on, participants wore an actigraph (MotionWatch 8; CamNtech; Table 1) and filled in daily online sleep protocols (LimeSurvey), which were checked for compliance with a regular sleep–wake rhythm. An overview of all key resources is given in Table 1.

10.1523/ENEURO.0259-23.2024.f1-1 Figure 1-1. Entrance questionnaire results (mean and standard deviation; *N* = 28). Download Figure 1-1, DOCX file.

**Figure 2.**
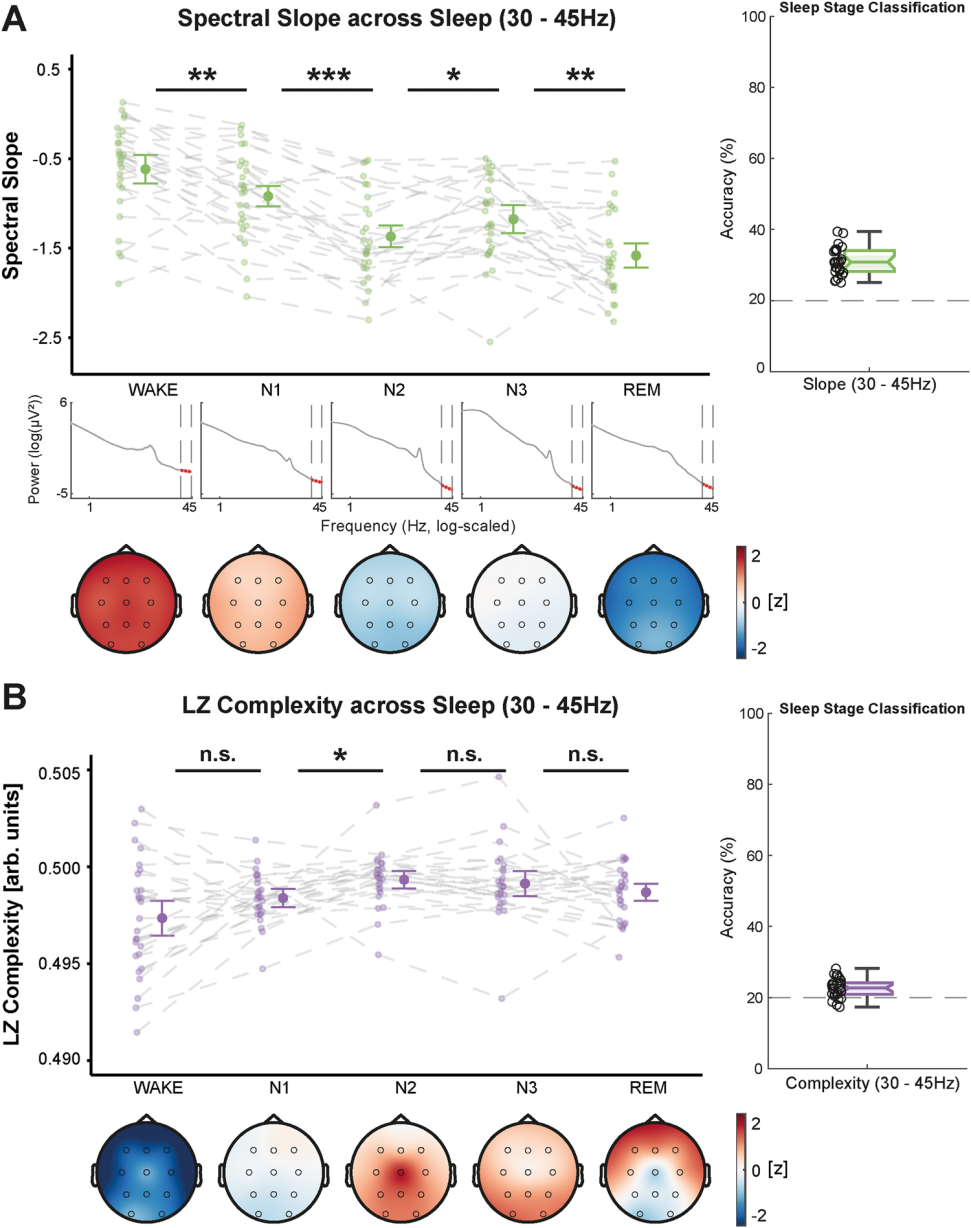
Spectral slope (green, ***A***) and Lempel–Ziv (LZ) complexity (purple, ***B***) from 30 to 45 Hz across sleep, averaged over all lab-sessions per subject. Center figures show the data averaged over all electrodes and topographical maps are provided below (color-coding refers to *z* values of slope or complexity computed from the grand average across all sleep stages). In ***A***, the log–log power spectra are provided for each sleep stage to illustrate the slope changes across different sleep stages. Classification accuracies are shown on the right side. ***A***, The spectral slope decreases from wakefulness across all sleep stages to REM sleep with a small temporary increase during N3 sleep. ***B***, Lempel–Ziv complexity increases from shallow N1 to light N2 sleep and is in general less modulated by sleep stage than the spectral slope. EMG activity did not confound the modulation of the spectral slope and Lempel–Ziv complexity during sleep (Extended Data Fig. 2-1) or wakefulness (Extended Data Fig. 2-2). ***p < 0.001, **p ≤ 0.010, *p ≤ 0.050, ^n.s.,^*p* > 0.050; all *p* values are adjusted for multiple comparisons; error bars represent 95% confidence intervals (*N* = 27).

10.1523/ENEURO.0259-23.2024.f1-2 Figure 1-2. Whole night sleep architecture for all lab visits (median and interquartile range; *N* = 28). Download Figure 1-2, DOCX file.

**Figure 3.**
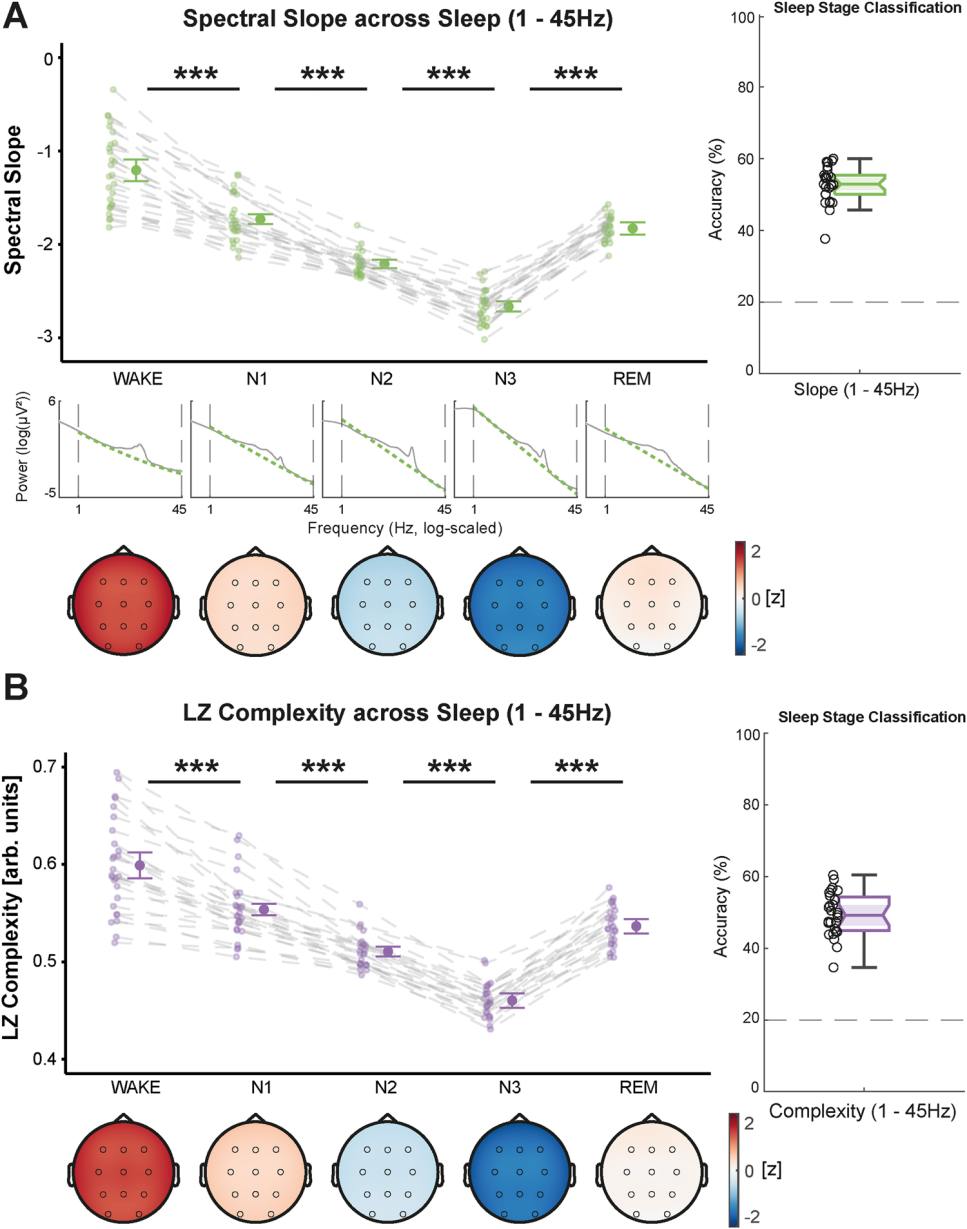
Spectral slope (green, ***A***) and Lempel–Ziv (LZ) complexity (purple, ***B***) from 1 to 45 Hz across sleep, averaged over all lab-sessions per subject. Center figures show the data averaged over all electrodes and topographical maps are provided below (color-coding refers to *z* values of slope or complexity computed from the grand average across all sleep stages). In ***A***, the log–log power spectra for each sleep stage are provided to illustrate the broadband slope differences across sleep stages. Classification accuracies are shown on the right side. ***A***, Spectral slope steepens from wakefulness to N3 sleep but flattens to some extent in REM sleep. ***B***, Lempel–Ziv complexity shows the same pattern as the spectral slope and likewise decreases from wakefulness to N3 with a subsequent increase in REM sleep. ***p < 0.001, **p ≤ 0.010, *p ≤ 0.050, ^n.s.^*p* > 0.050; *p* values are adjusted for multiple comparisons; error bars represent 95% confidence intervals (*N* = 27).

10.1523/ENEURO.0259-23.2024.f1-3 Figure 1-3. Mean number of clean epochs (min, max) for all tasks and sleep stages per experimental condition (i.e., different lab-visits). For the wakefulness recordings, the data is averaged over the multiple measurements per lab-visit and the encoding session has been pooled over both runs per visit (*N* = 28). Download Figure 1-3, DOCX file.

**Figure 4.**
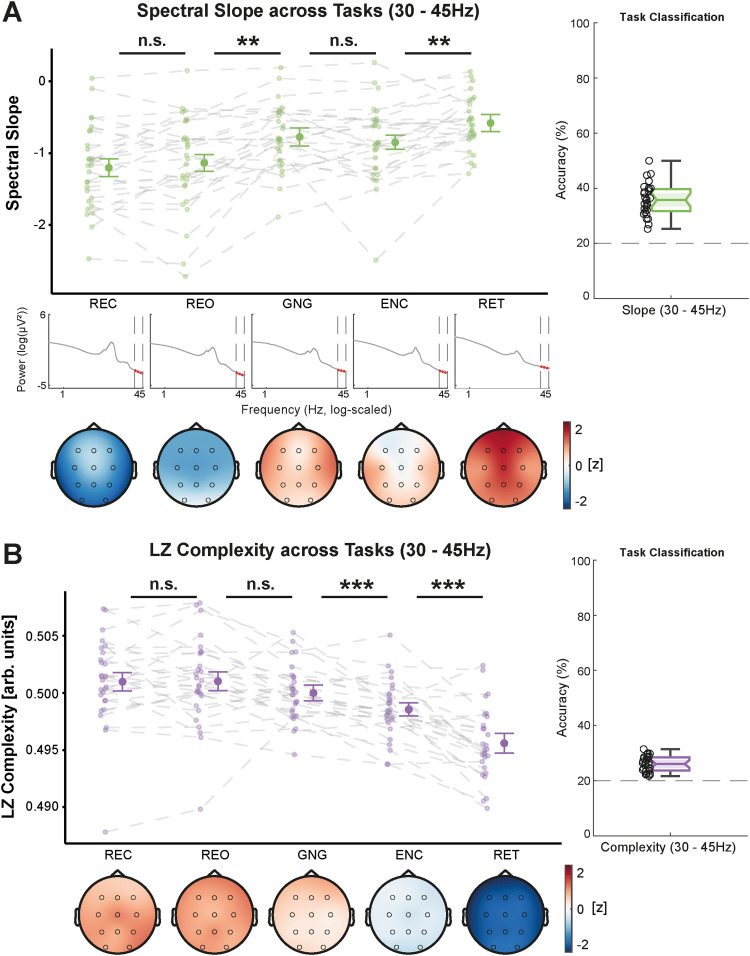
Spectral slope (green, ***A***) and Lempel–Ziv (LZ) complexity (purple, ***B***) from 30 to 45 Hz across tasks, averaged over all lab-sessions per subject (see Extended Data Fig. 4-1 for an analysis averaged over all timepoints per lab-session and Extended Data Fig. 4-2 for an analysis demonstrating similar results when using a different task order). Center figures show the data averaged over all channels and topographical maps are provided below (color-coding refers to *z* values of slope or complexity computed from the grand average across all tasks). In ***A***, the log–log power spectra for each task are provided to illustrate narrowband slope differences across tasks. Classification accuracies are shown on the right side. ***A***, The spectral slope flattens when engaging in cognitive tasks (Go/Nogo and learning) and is flattest during the retrieval session of the learning task. ***B***, Lempel–Ziv complexity decreases with task engagement and reaches its minimum during the retrieval session. ***p < 0.001, **p ≤ 0.010, *p ≤ 0.050, ^n.s.^*p* > 0.050; *p* values adjusted for multiple comparisons; error bars show 95% confidence intervals (*N* = 28).

10.1523/ENEURO.0259-23.2024.f1-4 Figure 1-4. Illustration of the effect of signal regularity on resulting Lempel-Ziv complexity values and the shape of their power-spectra. The complexity values increase from a binary boxcar signal (purple) to a pure 10 Hz alpha oscillation (blue), further to the same oscillation with additional pink noise (red) and to pure pink noise (orange). Completely random white noise (green) has the highest complexity. Download Figure 1-4, TIF file.

**Table 1.** Key resources

Reagent type (species) or resource	Designation	Source or reference	Identifiers	Additional information
Software, algorithm	BrainVision Analyzer 2.2	Brain Products	RRID: SCR_002356	https://www.brainproducts.com
Software, algorithm	Adobe Illustrator CS6	Adobe	RRID: SCR_010279	
Software, algorithm	FieldTrip (obob_ownft)	Oostenveld et al., 2011	RRID: SCR_004849	https://gitlab.com/obob/obob_ownft/
Software, algorithm	FOOOF (specparam)	Donoghue et al., 2020		https://fooof-tools.github.io/fooof/
Software, algorithm	ggplot-2	Wickham, 2016	RRID: SCR_014601	https://cran.r-project.org/web/packages/ggplot2/index.html
Software, algorithm	Lempel–Ziv complexity	Comsa, 2019		https://github.com/iulia-m-comsa/EEG/tree/master/Lempel-Ziv%20complexity
Software, algorithm	MANOVA.RM	Friedrich et al., 2019		https://cran.r-project.org/web/packages/MANOVA.RM/index.html
Software, algorithm	MATLAB 2018b	MathWorks	RRID: SCR_001622	https://de.mathworks.com/products/matlab.html
Software, algorithm	MVPA-light toolbox	Treder, 2020	RRID: SCR_022173	https://github.com/treder/MVPA-Light
Software, algorithm	Psychtoolbox PTB-3	Kleiner et al., 2007	RRID: SCR_002881	http://psychtoolbox.org/
Software, algorithm	RStudio 2021.09	RStudio Team	RRID: SCR_000432	https://posit.co/downloads/
Software, algorithm	Somnolyzer 24 × 7	Koninklijke Philips N.V.		https://www.philips.co.in

The first recording was scheduled on day 4 for adaptation purposes to avoid potential first night effects (Browman and Cartwright, 1980; Curcio et al., 2004). After electrode placement, participants were familiarized with the resting session and Go/Nogo (GNG) task. Bedtime was scheduled for ∼11:00 P.M., and participants were woken up 8 h after lights out, thereby adhering to general sleep hygiene recommendations (Watson et al., 2015; Chaput et al., 2018). The experimental recordings were scheduled on days 7, 10, and 13. Participants arrived at 6:00 P.M. and electrodes were mounted again. Recordings started with an initial resting session (3 min eyes closed and 3 min eyes open) and the GNG task (10 min), which was followed by the encoding sessions (two times 14 min) of a declarative memory task. Before the first cued recall, another resting and GNG session was conducted. Afterward, participants had a break of 1.5 h in which they read standardized stories under different light conditions. These conditions consisted of either reading from a smartphone with or without a blue light filter or from a printed book, leading to varying levels of short-wavelength light exposure with dim background room lighting (Höhn et al., 2021; Schmid et al., 2021). Before going to bed at ∼11:00 P.M., participants completed the last resting and GNG session of the day. After awakening, a morning session of resting and the GNG task as well as another cued recall session were performed. During all wake recordings, daylight mimicking room lights (provided by Emilum) were dimmed to 4.5 photopic lux, and room temperature was adjusted via air conditioning based on participant's preferences.

#### Go/Nogo task

To assess objective levels of attention and inhibitory control, we implemented an auditory version of the Go/Nogo paradigm (Donders, 1969) via the Psychophysics Toolbox (PTB-3; Kleiner et al., 2007) in MATLAB (Release 2018b, The MathWorks). Due to the plentitude of tasks already included in the study design, we opted for an attention task that measures various aspects of attention and inhibitory control and therefore chose the Go/Nogo paradigm over a classical psychomotor vigilance task. Thus, participants were asked to react as quickly as possible on a response time box (RTBox v5/6; Ohio State University) whenever they heard a “Go” sound and needed to inhibit their reaction when a “Nogo” sound was played. The task comprised 400 trials with Go sounds being presented in 80% of the trials (the order of Go and Nogo sounds was randomized each time). The two stimuli used for the Go and Nogo sounds were low- (1,000 Hz) and high-pitched (1,500 Hz) tones, which were presented for 50 ms with a varying interstimulus interval (1,480–1,880 ms). Whether the low- or high-pitched sound represented the Go-signal was determined by chance at the beginning of each session. Participants had to react within 500 ms for the response to be considered valid, and reaction times longer than 500 ms were regarded as attentional lapses. From each session, the performance score was computed by dividing the percentage of correct trials by the median reaction time of all valid responses (≤500 ms, no errors) in milliseconds (Figueiro et al., 2016).

#### Declarative memory task

Participants encoded a set of 80 word pairs on days 7, 10, and 13. To avoid learning effects over time, we presented a different but similarly difficult set of 80 word pairs on each of the 3 days. The order of the sets was randomized across subjects. Each set was presented twice for 14 min during encoding, and the data from both encoding sessions was pooled. Each word pair was presented for 1,500 ms, followed by a fixation-cross for 8,500 ms. Participants were instructed to encode the word pair as vividly as possible during the presentation of the fixation-cross by imagining a semantic connection between the two words. During the cued recall sessions, only the first word of a pair was presented, and participants were asked to press a button on the response time box as soon as they remembered the second word. Whenever a button was pressed, the participant was instructed to name the missing word and a fixation-cross appeared for 3,500 ms while the experimenter noted the answer. When no button was pressed, the fixation-cross appeared automatically after 6,500 ms. Recall performance was measured as the percentage of correctly recalled word pairs during each retrieval session. To assess the overnight change in performance, we computed the increase in percentage from the evening performance to the following morning.

### EEG recording and analyses

All electrophysiological data were recorded with a sampling rate of 500 Hz via the BrainVision Recorder software (Version 2.11) using a 32-channel BrainAmp system (Brain Products). We placed 11 gold-cup electrodes (Grass Technologies, Astro-Med) according to the international 10–20 system on the positions: F3, Fz, F4, C3, Cz, C4, P3, Pz, P4, O1, and O2. Linked mastoids were used for offline re-referencing as the data were online referenced against Cz. The position Fpz was used as ground electrode. Additionally, two EMG electrodes were placed on the musculus mentalis for measuring muscle activity during sleep and four EOG electrodes around the eyes to record horizontal and vertical eye movements. Impedances were always kept below 10 kΩ.

#### Polysomnography

Time in bed was standardized for all polysomnography recordings to 8 h. For sleep staging, the data were first low-pass filtered at 30 Hz and re-referenced to contralateral mastoids with the BrainVision Analyzer software (Version 2.2.0.7383, Brain Products GmbH, 2019). EOG and EMG channels were referenced bipolarly and the data were down-sampled to 128 Hz for further staging. Sleep stages were classified for each 30 s epoch with the Somnolyzer 24 × 7 algorithm (Koninklijke Philips N.V.) in accordance with the criteria of the American Academy of Sleep Medicine (Richard et al., 2012). The results were finally verified by a human expert scorer. The general sleep architecture of each night is presented descriptively in Extended Data Figure 1-2.

#### EEG preprocessing

In a first step, the raw data were processed with the BrainVision Analyzer software, and we applied a 0.3 Hz high-pass as well as a 50 Hz notch filter. EEG channels were re-referenced to linked mastoids and the online reference Cz was restored. We corrected for eye movements with the Gratton and Coles method (Gratton et al., 1983; only implemented for data during wakefulness) and ran an automatic artifact detection procedure on all scalp EEG channels, which was manually checked afterward. Events with a voltage jump exceeding 50 μV/ms, an absolute voltage difference of >400 μV within 200 ms or activity <0.5 μV for at least 100 ms were marked as bad intervals. If severe muscle or movement artifacts were missed, they were additionally marked manually. The data were then down-sampled to 250 Hz and exported for further analyses in MATLAB. The continuous data were subsequently segmented into epochs of 4 s for each task and sleep stage using the FieldTrip toolbox (Oostenveld et al., 2011). To be able to compare all task and sleep data, we decided to set the epoch length to 4 s as this enabled the best tradeoff between sufficient epochs even for the shortest tasks (3 min resting sessions) and an adequate frequency resolution. All artifact-containing epochs (defined as >1% being detected as artifact) were removed. Since the remaining number of clean epochs from the tasks (resting, Go/Nogo, encoding, and retrieval) and sleep stages (WAKE, N1, N2, N3, and REM) varied due to different recording lengths, we balanced the number of epochs across tasks and sleep stages for the multivariate pattern (MVPA) analyses. We set the maximum number of epochs for the MVPA analyses to the highest possible number of epochs from the shortest task (i.e., 45 epochs as the resting sessions only comprised 3 min). To do so, we drew a random subset of 45 epochs from all data that contained >45 clean epochs. For all other analyses, we used all available data to maximize the signal-to-noise ratio wherever possible (compare Extended Data Fig. 1-3).

#### Spectral slope

To obtain the spectral slope, we first calculated power spectra between 0.5 and 45 Hz from the preprocessed, 4 s segmented data via the *mtmfft* method in FieldTrip (Oostenveld et al., 2011) using a multitaper approach (1 Hz frequency smoothing). To extract the slope, we applied robust linear fits (using the *robust fit* MATLAB function) in log–log space between 30 and 45 Hz. We decided to use robust linear fits instead of using the *FOOOF* algorithm (Donoghue et al., 2020) for the narrowband frequency range since this approach has already been established to yield a sensitive aperiodic marker of arousal by Lendner et al. (2020) and because in this frequency range also the *FOOOF* would approximate a linear fit, thus leading to highly comparable results. However, for the broadband frequency range (1–45 Hz), we applied the *FOOOF* algorithm to extract the slope since linear fits would have been skewed by oscillatory bumps in the power spectrum.

#### Lempel–Ziv Complexity

We followed previous approaches (Schartner et al., 2015; Mediano et al., 2021; Medel et al., 2023) and calculated the Lempel–Ziv-Welch complexity (Lempel and Ziv, 1976; Welch, 1984) per channel and epoch. To obtain the complexity in the same frequency ranges in which we calculated the spectral slope, we applied additional 1 or 30 Hz high-pass and 45 Hz low-pass filters. As Rivolta et al. (2014) demonstrated that 1,000 datapoints are sufficient for reliable Lempel–Ziv complexity analyses during sleep, we used the same 4 s segmented data (corresponding to 1,000 sampling points per epoch in our down-sampled data) as for the spectral slope. We then applied a Hilbert transformation on each epoch to obtain the instantaneous amplitude. Afterward, we binarized the resulting single epoch data around its median amplitude and transformed it into a binary sequence. Values of 1 were given for amplitude samples above the median and values of 0 for amplitudes below (or equal with) the median. This binary sequence of ones and zeros was subjected to the Lempel–Ziv–Welch complexity algorithm (Comsa, 2019) in MATLAB. In general, higher complexity values (normalized between 0 and 1) reflect more random and unpredictable signals (compare Extended Data Fig. 1-4).

### Statistical analyses

Statistics were calculated in RStudio (Version 4.1.2.). MATLAB functions from the FieldTrip toolbox and the ggplot-framework (Wickham, 2016) in R were adapted for data visualization.

#### ANOVA-type analyses and correlations

All analyses involved three repeated measurements (days 7, 10, and 13; compare Fig. 1) and therefore at least two factors (lab-session and task or sleep stage). Since in most cases at least one assumption for parametrical testing was violated, we decided to compute more conservative semiparametrical analyses with the MANOVA.RM package (Friedrich et al., 2019). For these factorial analyses, data were averaged over all EEG electrodes to facilitate interpretation of the results. In the statistical results, we always refer to the Wald-type statistics (WTS) with empirical *p* values obtained from permutation resampling procedures and 10,000 iterations. Whenever multiple comparisons were conducted, *p* values were corrected with the Benjamini–Hochberg procedure (Benjamini and Hochberg, 1995).

For correlational analyses, we computed the Spearman rho coefficients instead of Pearson’s correlations whenever the normality assumption was significantly violated (indicated by Shapiro–Wilk tests) and for all cluster correlations on the whole scalp level. For the cluster corrected correlation approach, we used the Monte Carlo method with 10,000 iterations to assess the relationship between EEG parameters per channel and behavioral measures.

#### Multivariate pattern analyses

Since it is difficult to take topographical patterns into account in classical factorial designs, we additionally computed MVPA analyses using the MVPA-Light toolbox (Treder, 2020) to exploit the information present in the complexity and slope data as patterns across electrodes. For each task and sleep stage, the complexity and slope from every epoch and electrode was fed into the classifier. Thus, the single epochs per subject were used for training and testing while the complexity and slope patterns over electrodes represented the multivariate information. For comparisons between more than two tasks or sleep stages, multiclass linear discriminant analyses (LDAs) were used and regular LDA for two-condition comparisons. We calculated classification accuracies per subject via leave-one-out cross-validation (LOO-CV) to account for the restricted amount of data available for training and testing. Since no effects regarding the different lab-sessions emerged, we pooled the data from the different lab-sessions for each participant to improve the reliability of the MVPA analyses.

### Data and code accessibility

The data and code necessary to reproduce the main results and figures is freely available online at https://doi.org/10.17605/OSF.IO/QGPW4.

## Results

We calculated the spectral slope and Lempel–Ziv complexity for all sleep stages and tasks in a narrowband (30–45 Hz) and broadband (1–45 Hz) frequency range (compare Fig. 1*B,C*; Colombo et al., 2019; Lendner et al., 2020, 2022; Jacob et al., 2021; Kozhemiako et al., 2022; Ameen et al., 2023; Helson et al., 2023). We set the upper frequency limit to 45 Hz to avoid line-noise influences ∼50 Hz and the need for fitting a knee in higher frequencies. Likewise, we decided to set the lower frequency boundary for the broadband range at 1 Hz since the area below often exhibits a plateau that would require fitting an additional knee or would otherwise also distort the data (He et al., 2010; Gerster et al., 2022). The 30 Hz lower limit for the narrowband range was selected based on original modeling work for the spectral slope, demonstrating that the 30–45 Hz range is capable of tracking changes in the brain's excitation/inhibition balance (Gao et al., 2017). Furthermore, a large replication study with >10,000 polysomnography recordings confirmed that the 30–45 Hz range tracks the hypnogram accurately (Kozhemiako et al., 2022), a finding that has also been supported by others who used a data-driven fitting algorithm to compare different frequency ranges for slope estimation (Lendner et al., 2020, 2022).

### Spectral slope and Lempel–Ziv complexity delineate brain states during sleep

The narrowband slope and complexity were significantly modulated by sleep stage (N1, N2, N3, and REM sleep: WTS_(4)_ = 133.57, *p* < 0.001 and WTS_(4)_ = 21.64, *p* = 0.004, respectively). The narrowband slope was significantly steeper in all sleep stages compared with wakefulness with the steepest slope during REM sleep. In contrast, the narrowband complexity slightly increased from wakefulness to all sleep stages (Fig. 2). To control for muscular activity in the 40–70 Hz range, we also computed the slope and complexity of the EMG channels (Extended Data Figs. 2-1, 2-2). When partialling out the EMG from the EEG data, the modulation of the EEG slope and complexity remained largely unaffected, indicating that EMG activity did not significantly confound the results.

10.1523/ENEURO.0259-23.2024.f2-1 Figure 2-1. Control analyses including the narrowband spectral slope from the EMG. A: The negative correlations between EEG slope and sleep stage do not change when partialling out the EMG slope. B: While the average EEG slope is negatively correlated with sleep stage, the EMG slope is even slightly positively correlated with sleep stage and significantly different from the EEG slope correlation. C: The positive correlations between EEG slope and the cognitive tasks (ordered ascendingly regarding their slope) are not diminished when controlling for the EMG. D: While the correlation between the EMG slope and the tasks is slightly higher than between the EEG slope and the tasks, partialling out the EMG from the EEG slope does not significantly reduce the correlation. E & F: Differential modulation of the EEG & EMG slopes across sleep stages and tasks. Download Figure 2-1, TIF file.

10.1523/ENEURO.0259-23.2024.f2-2 Figure 2-2. Control analyses including the narrowband Lempel-Ziv complexity (LZC) from the EMG. A: The positive correlations between EEG complexity and sleep stage do not change when partialling out the EMG complexity. B: While both, the average EEG and EMG complexity are positively correlated with sleep stage, the partial correlation controlling for EMG complexity does not shrink substantially. C: The negative correlations between EEG complexity and the cognitive tasks are not changed substantially by partialling out the EMG. D: Both, the average EEG and EMG complexity are negatively correlated with the tasks during wakefulness but the partial correlation between EEG complexity and the tasks controlled for the EMG is not significantly smaller. E & F: Differential modulation of the EEG & EMG complexity across sleep stages and tasks. Download Figure 2-2, TIF file.

When the broadband frequency range was used for estimation, the effect of sleep stage was even more pronounced in both parameters (spectral slope: WTS_(4)_ = 1,088.28, *p* < 0.001; Lempel–Ziv complexity: WTS_(4)_ = 857.60, *p* < 0.001). Both, broadband slope and complexity, significantly decreased from shallow (N1) to deep NREM sleep (N3). For REM sleep, however, both markers increased again (Fig. 3), arguably reflecting more wake-like brain activity. While REM sleep was significantly different from wakefulness in both frequency ranges for both parameters (all *p* < 0.025), it was best discernable with the narrowband slope. We found no significant effects of the repeated measurements (all *p*_adj._ ≥ 0.419), revealing that the effect of sleep stage robustly emerged in all individual recordings per subject.

### Spectral slope and Lempel–Ziv complexity vary across tasks

Next, we investigated whether spectral slope and Lempel–Ziv complexity can differentiate between resting and task engagement as well as between the different active tasks. We calculated both markers from resting sessions with eyes closed (REC) and eyes open (REO), an auditory Go/Nogo (GNG) task, an encoding session (ENC) from a declarative memory task, as well as its retrieval session (RET). For this, we focused on the task data from the evening recordings (see dashed dark-green rectangle in Fig. 1*A*). Theoretically, a task engagement effect, representing a shift toward excitation (i.e., flatter slopes and higher complexity), should be visible between the resting sessions and the GNG or learning task. Since the GNG task was mainly auditory and should rely on different cognitive resources compared with the visual/verbal memory task, we also expected differences between the GNG, ENC, and RET sessions.

In the narrowband range, we observed a significant flattening of the slope (WTS_(4)_ = 56.64, *p* < 0.001) along with a decrease in complexity (WTS_(4)_ = 199.55, *p* < 0.001) from resting sessions to active tasks, including GNG, ENC, and RET (Fig. 4). As expected, the slope was flatter during the GNG, ENC, and RET tasks than during resting. However, there was an additional flattening of the narrowband slope during RET compared with that during GNG and ENC, potentially reflecting higher cognitive engagement. Narrowband Lempel–Ziv complexity did not differ between the resting and GNG sessions (all *p*_adj._ > 0.110) but decreased from the GNG to the ENC session and was lowest during retrieval.

10.1523/ENEURO.0259-23.2024.f4-1 Figure 4-1. Slope and complexity (30 – 45Hz) across tasks averaged over all timepoints. Download Figure 4-1, TIF file.

10.1523/ENEURO.0259-23.2024.f4-2 Figure 4-2. Slope and complexity (30 – 45Hz) across tasks using a different task-order (REC#1, GNG#1, ENC, REO#2, RET#1 instead of ENC, REC#2, REO#2, GNG#2, RET#1, cf., Figure 1). Download Figure 4-2, TIF file.

**Figure 5.**
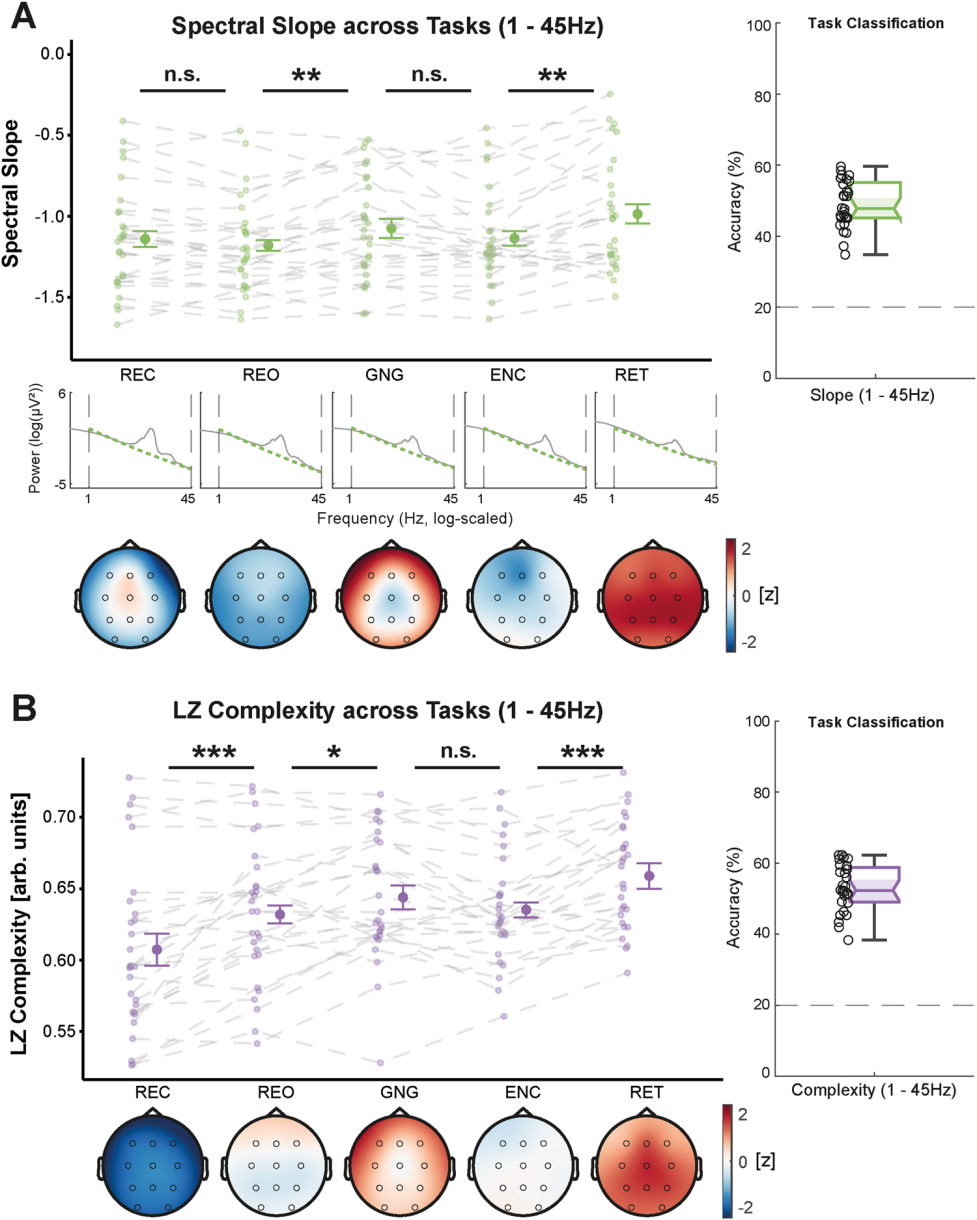
Spectral slope (green, ***A***) and Lempel–Ziv (LZ) complexity (purple, ***B***) from 1 to 45 Hz across tasks, averaged over all lab-sessions per subject (see Extended Data Figs. 5-1 and 5-2 for analyses averaged over all timepoints per session and for a different task order). Center figures show the data over all channels and topographical maps are provided below (color-coding refers to *z* values of slope or complexity computed from the grand average over all tasks). In ***A***, the log–log power spectra for each sleep stage are provided to illustrate broadband slope differences across tasks. Classification accuracies are shown on the right side. ***A***, The slope flattens from resting to the Go/Nogo task (see Extended Data Fig. 5-3 for an analysis of the effect of different epoch types and lengths for this task) and is flattest during retrieval. ***B***, Complexity increases from resting with closed to open eyes and is further elevated in all active tasks, peaking during retrieval. ***p < 0.001, **p ≤ 0.010, *p ≤ 0.050, ^n.s.^*p* > 0.050; *p* values adjusted for multiple comparisons; error bars show 95% confidence intervals (*N* = 28).

When investigating the broadband frequency range, we found that the diverging pattern between spectral slope and Lempel–Ziv complexity disappeared and both parameters were increasing from rest to active task engagement (slope: WTS_(4)_ = 40.46, *p* < 0.001; complexity: WTS_(4)_ = 46.24, *p* < 0.001; Fig. 5). In addition, Lempel–Ziv complexity differed between the two resting sessions (eyes closed and eyes open), likely reflecting a difference in alpha power (8–12 Hz). This further supports the notion that oscillatory components exert a greater influence on Lempel–Ziv complexity than those on the spectral slope. Again, we did not observe any effects of the repeated measurements (all *p*_adj._ ≥ 0.222).

10.1523/ENEURO.0259-23.2024.f5-1 Figure 5-1. Slope and complexity (1 – 45Hz) across tasks averaged over all timepoints. Download Figure 5-1, TIF file.

10.1523/ENEURO.0259-23.2024.f5-2 Figure 5-2. Slope and complexity (1 – 45Hz) across tasks using a different task-order (REC#1, GNG#1, ENC, REO#2, RET#1 instead of ENC, REC#2, REO#2, GNG#2, RET#1, cf., Figure 1). Download Figure 5-2, TIF file.

10.1523/ENEURO.0259-23.2024.f5-3 Figure 5-3. Effect of choosing 1s epochs around a stimulus (GO or NOGO) or 1s epochs during an interstimulus interval (ISI) or 4s epochs across the whole task. While the general epoch-length has a strong impact on the complexity estimates (C – D), neither the slope nor the complexity is strongly affected by different epoch-types (A – D). Download Figure 5-3, TIF file.

To control for task order and potential influences of exhaustion, we repeated the analyses with the task data averaged over all timepoints (compare Fig. 1*A*; i.e., the resting and GNG data were averaged over four timepoints, the RET data were over two timepoints, and the ENC was only completed once) and with a different order of tasks. Both control analyses showed the same pattern as the original analysis (compare Extended Data Figs. 4-1, 4-2 as well as Extended Data Figs. 5-1, 5-2). Further, we tested in the GNG task whether it would have made a difference if we focused only on epochs with or without stimulus presentation and did not detect strong differences between different epoch types (compare Extended Data Fig. 5-3).

**Figure 6.**
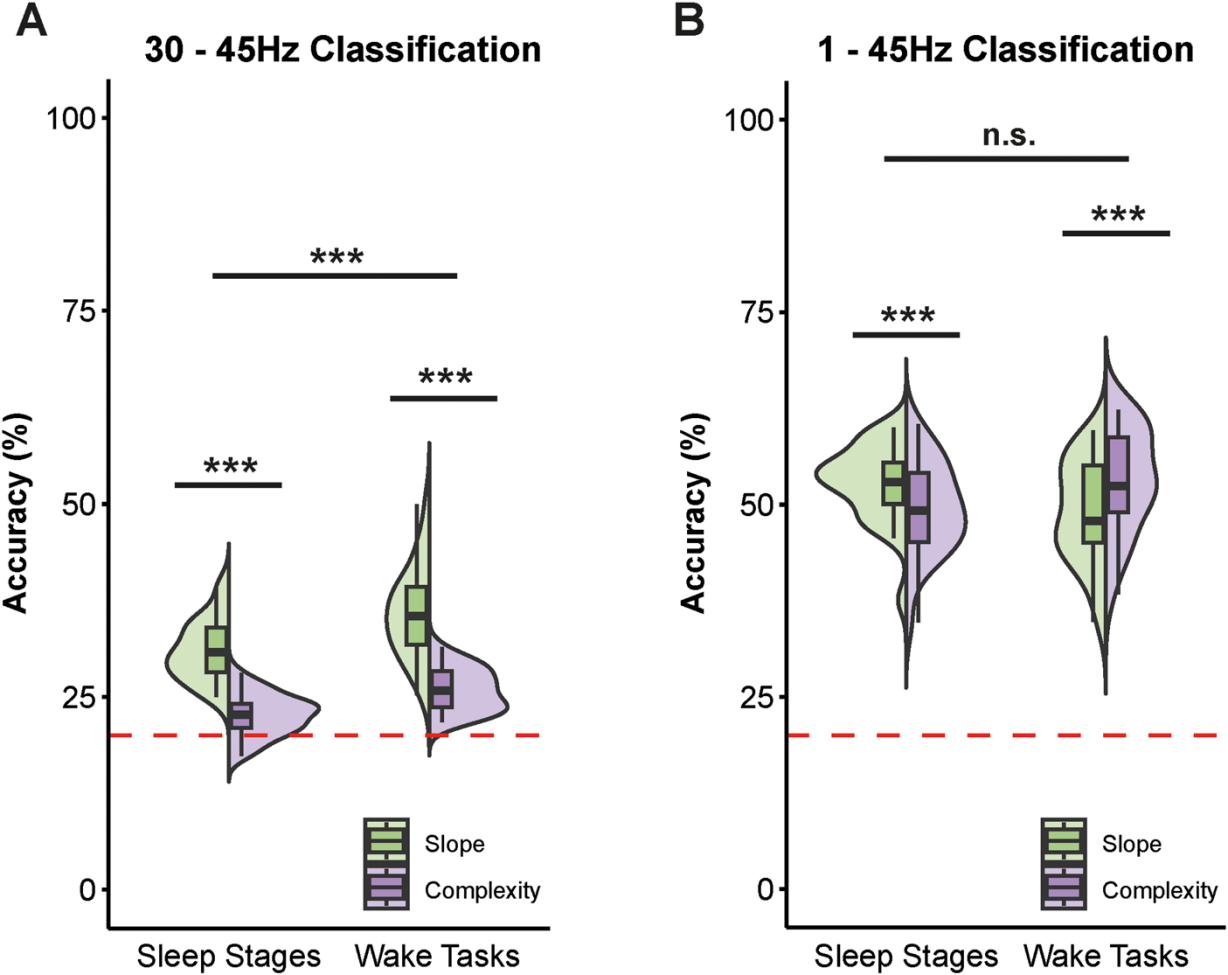
***A***, Comparison of the multiclass classification accuracies within 30–45 Hz (see Extended Data Fig. 6-1 for all pairwise classification accuracies) for the spectral slope and Lempel–Ziv complexity regarding sleep stages (WAKE, N1, N2, N3, REM) and tasks during wakefulness (resting eyes closed, resting eyes open, auditory Go/Nogo, encoding, retrieval). Sleep stages and tasks could be decoded more precisely with the spectral slope. Overall classification accuracy was significantly higher for tasks than for sleep stages. ***B***, Comparison of the classification accuracies across tasks and sleep stages for slope and complexity within 1–45 Hz (see Extended Data Fig. 6-2 for the pairwise classifications). The slope only yielded better decoding performance during sleep, whereas task classification worked better when using Lempel–Ziv complexity, which is arguably due to the difference in complexity between the two resting conditions that is not present in the slope. The dotted red lines represent chance level (20%). The correlation between slope and complexity within 30–45 Hz and 1–45 Hz is presented in Extended Data Figure 6-3. Extended Data Figures 6-4 and 6-5 demonstrate the robustness of the slope and complexity values across lab-visits and the correlation across frequency ranges. ***p < 0.001, ^n.s.^*p* > 0.050 (*N *= 28).

To evaluate the topographical distribution of the spectral slope and Lempel–Ziv complexity, we also ran MVPA analyses with multiclass LDAs. Thus, we quantified how well sleep stages and tasks could be decoded by taking the topographical distribution of the slope and complexity into account. In both frequency ranges and for both parameters, classification accuracies were significantly above chance level (20%; *p* < 0.001). In the narrowband range, the slope was significantly more informative about the underlying brain state (i.e., yielded higher classification accuracies) than complexity (sleep: WTS_(1)_ = 166.15, *p* < 0.001; wake: WTS_(1)_ = 82.28, *p* < 0.001; compare Fig. 6*A*). Within the broadband range, this was only true for the sleep stage classification (WTS_(1)_ = 19.84, *p* < 0.001). As for the classification of tasks during wakefulness, the Lempel–Ziv complexity was more informative (WTS_(1)_ = 22.38, *p* < 0.001; compare Fig. 6*B*). An overview of the pairwise classification accuracies for all sleep stages and task pairings is presented in Extended Data Figures 6-1 and 6-2. Correlations between slope and complexity as well as analyses of the robustness across repeated measurements are presented in Extended Data Figures 6-3, 6-4, and 6-5.

10.1523/ENEURO.0259-23.2024.f6-1 Figure 6-1. Classification accuracy for all pairwise combinations of sleep stage and task (30 – 45Hz). Upper triangular matrix shows the results for Lempel-Ziv complexity and lower triangular matrix for the spectral slope. Data was pooled over all lab-visits for each subject. Download Figure 6-1, TIF file.

10.1523/ENEURO.0259-23.2024.f6-2 Figure 6-2. Classification accuracy for all pairwise combinations of sleep stage and task (1 – 45Hz). Upper triangular matrix shows the results for Lempel-Ziv complexity and the lower triangular matrix for the spectral slope. The data was pooled over all lab-visits for each subject. Download Figure 6-2, TIF file.

10.1523/ENEURO.0259-23.2024.f6-3 Figure 6-3. Correlations between spectral slope and Lempel-Ziv (LZ) complexity from 30 – 45Hz and 1 – 45Hz. The sleep (A) and task (B) data per subject were averaged across all lab-sessions. For task data, only the evening assessments highlighted by the dashed dark-green rectangle in Figure 1 were considered. Significant correlations (*p* ≤ .050 after correcting for false discovery rate) are highlighted with a cross on the topographical maps (color codes for the size and directionality of the correlation coefficients). Download Figure 6-3, TIF file.

10.1523/ENEURO.0259-23.2024.f6-4 Figure 6-4. Robustness of the spectral slope and Lempel-Ziv complexity across lab-visits. Correlation coefficients over all electrodes for each parameter between the three experimental recordings (1 x 2, 1 x 3 and 2 x 3). Each of the experimental recordings refers to one lab-visit per subject. Download Figure 6-4, TIF file.

10.1523/ENEURO.0259-23.2024.f6-5 Figure 6-5. Correlation of the slope and complexity with themselves in the narrow- or broadband frequency range during sleep (A) and wakefulness (B). Download Figure 6-5, TIF file.

### The narrowband spectral slope as an electrophysiological marker of task performance

Having established that slope and complexity are not only modulated by sleep but also differ between tasks in a frequency range-specific manner, we next investigated their relationship with task performance. We correlated the slope and complexity from the narrow- and broadband frequency range during the GNG task with the according performance scores (percentage of correct trials divided by median reaction time) over multiple sessions.

**Figure 7.**
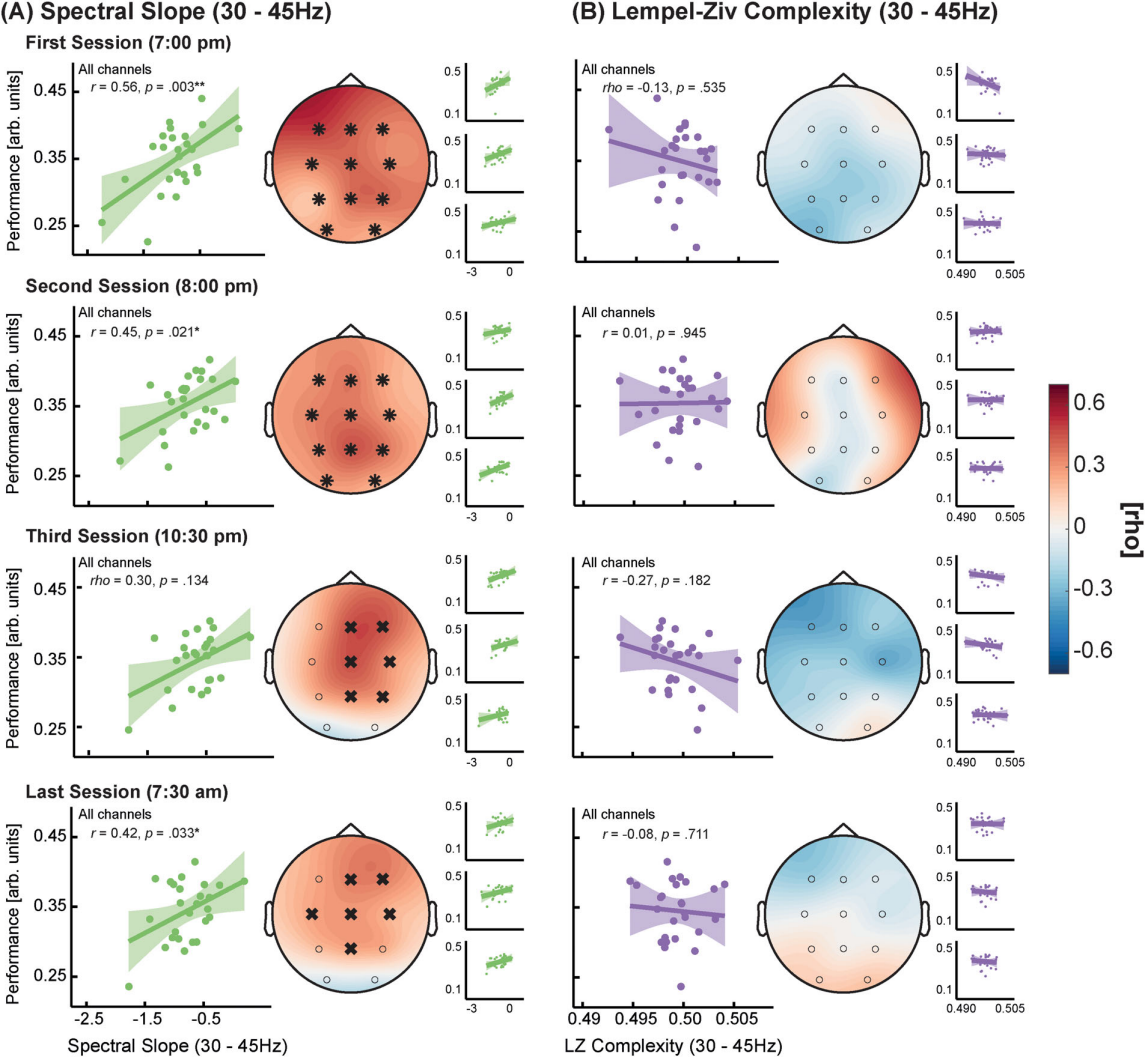
Relationship between Go/Nogo task performance and spectral slope (***A***) or Lempel–Ziv (LZ) complexity (***B***) within 30–45 Hz across different assessment times (see Extended Data Fig. 7-1 for the 1–45 Hz range). For the large scatterplots, data were averaged across all lab-sessions (small scatterplots show the relationship per lab-session). The topoplots depict the correlation strength for each electrode. Electrodes forming a significant cluster are highlighted with asterisks. Those showing a significant correlation after false discovery rate correction but did not from a cluster are marked with a cross. Only the narrowband spectral slope showed a consistent positive relationship with task performance (*N* = 26).

Within the narrowband range, the slope was more consistently related to higher task performance than the complexity, reaching statistical significance at 3 out of 4 timepoints (compare Fig. 7). In the broadband range, the relationship with task performance was also consistently positive for both parameters but did not reach statistical significance (compare Extended Data Fig. 7-1). Thus, only the narrowband slope reliably predicted GNG task performance.

10.1523/ENEURO.0259-23.2024.f7-1 Figure 7-1. Results when using the broadband (1 – 45Hz) frequency range. No significant relationships emerged for the spectral slope and Lempel-Ziv complexity, even though correlations were consistently positive for both parameters. Download Figure 7-1, TIF file.

**Figure 8.**
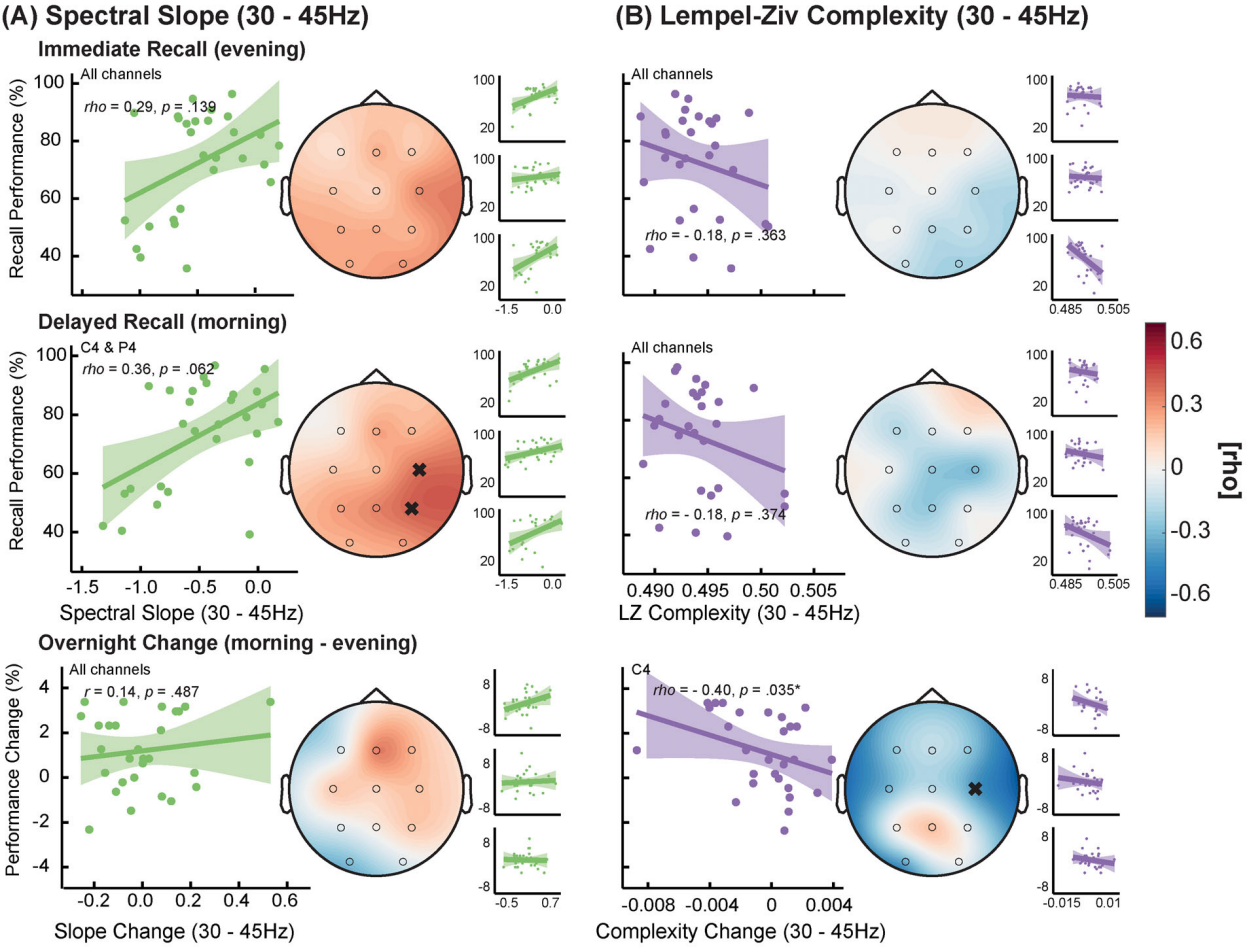
Relationship between declarative memory recall performance and spectral slope (***A***) or Lempel–Ziv (LZ) complexity (***B***) within 30–45 Hz (see Extended Data Fig. 8-1 for the 1–45 Hz range). Results are shown for immediate recall in the evening and delayed recall on the next morning as well as for overnight change. For the large scatterplots, data was averaged across all lab-sessions (small scatterplots show the relationship per session). The topoplots represent the strength of the correlations on each electrode. Even though the spectral slope was consistently positively correlated with recall performance, no electrodes formed a significant cluster. Significant single electrodes that survived false discovery rate correction are highlighted with a cross (*N* = 28).

Next, we determined whether the narrowband slope would also track memory performance. Thus, we correlated slope and complexity during the RET sessions of the declarative memory task with recall performance (i.e., percentage of correctly recalled word pairs). Even though the slope was again consistently positively correlated with recall performance, only few correlations (C4 and P4 at the delayed retrieval) reached statistical significance (Fig. 8). Despite the lack of statistical significance, the positive trend of tracking recall performance was only observed for the narrowband spectral slope. In the broadband frequency range, both parameters did not show a consistent relationship with recall performance (Extended Data Fig. 8-1).

10.1523/ENEURO.0259-23.2024.f8-1 Figure 8-1. Results when using the broadband 1 – 45Hz frequency range. No relationship observable between recall performance and slope or complexity. Download Figure 8-1, TIF file.

Finally, we assessed whether the performance in the GNG and RET task were related, resulting from better overall attention and higher task engagement. However, there was no significant relationship between the performance scores in the two tasks (evening: rho = 0.10, *p* = 0.611; morning: rho = 0.06, *p* = 0.766). Thus, subjects that performed well in the GNG task did not necessarily achieve high recall performance scores.

## Discussion

In this study comprising three experimental recordings with multiple measurements per subject, we demonstrated that the spectral slope and Lempel–Ziv complexity (1) reliably delineate sleep stages and (2) are modulated by different cognitive tasks. Critically, we provided evidence that (3) the modulation of the slope and complexity strongly depends on the frequency content. While the broadband (1–45 Hz) slope and complexity were more strongly modulated by sleep stages in general, the narrowband (30–45 Hz) slope best differentiated REM sleep from wakefulness and reflects mainly aperiodic activity. Moreover, we found that (4) active task engagement (i.e., switching from resting to an attention or learning task) was associated with flatter slopes in the narrow- and broadband range, but only with higher complexity in the broadband range. The broadband range was also better suited to capture differences between tasks in the classification analyses. However, (5) only the narrowband slope tracked task performance in an auditory attention task and trended toward significance regarding memory performance.

### The narrowband slope uniquely tracks aperiodic brain activity in REM

Our findings corroborate previous research demonstrating that the spectral slope and Lempel–Ziv complexity are sensitive markers of sleep stage (Abásolo et al., 2015; Schartner et al., 2017; Lendner et al., 2020; Bódizs et al., 2021; Kozhemiako et al., 2022; Pascovich et al., 2022). We extended these findings by leveraging repeated EEG recordings per subject and confirmed that the two parameters can robustly differentiate between all sleep stages and wakefulness across multiple recording days. In contrast to complexity, the calculation of the slope was ∼3.5 times more computationally efficient with our implementations, highlighting its practical applicability for closed loop and clinical settings.

Overall, sleep stages could be better delineated within a broadband frequency range. This is probably because the broadband range encompasses the frequencies typically used for traditional sleep scoring, such as slow wave activity (0.5–4 Hz) and sleep spindles (11–15 Hz; Dijk, 1995), thereby increasing the sleep stage-specific information in the underlying signal. Interestingly, REM sleep was only clearly distinguished from all other sleep stages by the narrowband slope, in line with findings from Lendner et al. (2020). In the broadband range, both parameters showed a relative, more wake-like, increase during REM sleep. Since REM sleep (sometimes called “paradoxical sleep”; Peigneux et al., 2001 or Siegel, 2011) is characterized by a more desynchronized EEG pattern that lacks prominent oscillations (Peever and Fuller, 2017; Blumberg et al., 2020), these disparate results between the two frequency ranges support the notion that the narrowband slope mainly measured aperiodic activity. The relative increase in broadband complexity during REM has been attributed to higher levels of conscious content that accompany vivid dreaming and thus require more complex brain activity than deeper, mostly dreamless sleep stages (Mateos et al., 2018; Lau et al., 2022).

Previous modeling work has linked the narrowband slope with the E/I balance in the brain (Gao et al., 2017). Within this framework, steeper slopes during REM sleep potentially reflect stronger inhibitory brain activity. This might allow the brain to decouple from its environment and, by maintaining muscle atonia, to enable the consolidation of emotional memories and the experience of vivid dreams (Aime et al., 2022) without acting them out. The narrowband complexity, however, does not appear to yield informative results and stayed almost constant across sleep stages. Thus, for complexity it might not be sensible to select a narrowband frequency range. Other research also provided evidence that complexity changes are mainly driven by frequencies at the lower end of the frequency spectrum (González et al., 2022).

### Aperiodic brain activity tracks task engagement and performance

We demonstrated that the spectral slope and Lempel–Ziv complexity can also track different tasks and are affected by task engagement. That slope and complexity are modulated during wakefulness is in line with other research (Sheehan et al., 2018; Jacob et al., 2021; Mediano et al., 2021; Waschke et al., 2021). However, to our best knowledge this is the first study comparing multiple tasks and different rest conditions as well as the effect of different frequency ranges. Like for sleep, we observed a homogenous modulation of the broadband slope and complexity, where flatter slopes and higher complexity values were associated with active task engagement.

In the E/I balance framework, flatter slopes are the result of higher excitation in the brain (Gao et al., 2017; Chini et al., 2022). Thus, our observed pattern of a flattening of the slope with task engagement and between cognitive tasks might be attributed to differences in the amount of required cognitive resources, leading to stronger excitatory brain activity (Harris and Thiele, 2011; He, 2011; Kanashiro et al., 2017). Unlike Waschke et al. (2021), who reported a stronger occipital flattening of the slope in a visual compared with an auditory task, we did not observe clear topographical differences between the auditory Go/Nogo task and the declarative memory task that relied on visual content. However, this lack of topographical distinctiveness might be due to a partial overlap of involved brain areas since both, auditory discrimination and learning, involve frontotemporal brain regions (Ackerman, 1992; Halsband, 1998).

When relating slope and complexity to behavior, we observed that only the narrowband slope was consistently correlated with attentional task performance across almost all recordings per subject (with the exception of the third session, compare Fig. 7). This association between flatter narrowband slopes and better task performance might even translate to cognitive tasks that do not solely rely on attention since we also observed a consistent but weaker and not statistically significant relationship with memory performance. However, in larger-scale studies which offer more reliable effect sizes and confidence intervals, the broadband slope and complexity were also significantly correlated with task performance (Mediano et al., 2021; Waschke et al., 2021). Taken together, our findings suggest that the narrowband slope serves as a particularly sensitive marker for task-dependent fluctuations in brain states relevant for behavioral performance.

### Narrow- and broadband frequency ranges track different facets of brain activity

Based on the results from the broadband range, it is tempting to assume that the spectral slope and Lempel–Ziv complexity are indexing similar features of brain activity. Indeed, according to Medel et al. (2023), both parameters might be driven by the transition entropy of the underlying cortical system (i.e., the predictive capacity of the current signal for the upcoming signal). Thus, flatter slopes and higher complexity values could be characteristic of the same cortical states. However, the divergence between the slope and complexity in the narrowband range clearly demonstrates that the two parameters are not redundant and indeed track different facets of the underlying signal. Different contributions of oscillatory and aperiodic brain activity might account for the diverging patterns. At first, it appears paradoxical that flatter slopes, representing an increase in aperiodic activity, should be accompanied by a decrease in complexity as complexity should also increase with higher irregularity. However, others have also reported this type of counterintuitive behavior. For instance, Mediano et al. (2021) showed that in MEG within 0.5–30 Hz, active tasks exhibited lower complexity values than quiet wakefulness. Additionally, a recent review from Lau et al. (2022) discussed several studies that reported contradicting modulations of signal complexity in different clinical conditions, where some report lower and others higher levels of complexity. So far, these contradictory findings seem to be best explained by the notion that higher complexity values can represent both, either more complex or more random systems (de la Torre-Luque et al., 2016), which makes it difficult to argue whether higher complexity always represents a healthier brain.

### Limitations

In this study, we focused exclusively on two specific EEG derivatives: spectral slope and Lempel–Ziv complexity. Consequently, we did not compare these measures against other established analytical techniques such as spectral power, entropy, or network analyses (e.g., coherence). However, regarding spectral power, previous research demonstrated that the spectral slope is superior in discriminating REM sleep from wakefulness compared with slow oscillation or gamma power and performs similarly to beta power (Lendner et al., 2020; Kozhemiako et al., 2022). Additionally, Biggs et al. (2022) showed that Lempel–Ziv complexity also performed similarly well in comparison with alpha power and outperformed permutation entropy in capturing age-related changes in brain activity induced by anesthesia. Nevertheless, comparisons with other biomarkers, such as heart rate variability or blood pressure (Radha et al., 2019; Mitsukura et al., 2020; Kuula and Pesonen, 2021), as well as specific benchmark tests involving both spectral slope and Lempel–Ziv complexity, are still lacking and merit exploration in future large datasets.

It should also be noted that the tasks in this study were not specifically designed for the analysis of varying levels of task demand or difficulty as the dataset was originally designed for other purposes. Even though the participants reported differences in task difficulty, other significant factors that could potentially influence the results include differences in task modality. While there is evidence that attentional and learning tasks do differ regarding their level of cognitive demand (Sweller, 2011; Bambrah et al., 2019), it also seems to be dependent on the specific task instructions and modalities. In the future, it might be promising to contrast tasks that exclusively rely on different cognitive resources and sensory modalities.

Despite using only 11 scalp electrodes and no high-density EEG caps due to the need for long-term EEG data collection (14–16 h), we still robustly detected modulations by sleep stage and task engagement, thereby providing evidence for the practical usability of the slope and complexity as indices of different brain states. Nevertheless, research with high-density or intracranial EEG might further contribute to the understanding of which topographical areas are most influential in driving changes in slope or complexity across brain states.

Finally, we only recruited healthy biologically male adults in a restricted age range (18–25 years) to avoid potential sex differences and hormonal effects (Plamberger et al., 2021; Kozhemiako et al., 2022) and because controlling for hormonal variations by exclusively recording female participants in the follicular phase would have been extremely challenging with our study design. Therefore, it is unclear to what extent our results generalize to other populations.

### Conclusions

Our results demonstrate that EEG spectral slope and Lempel–Ziv complexity are powerful indices of brain states during sleep and wakefulness. We present robust evidence from multiple recordings of three within-subject measurements, revealing that sleep stages and various cognitive tasks are reliably indexed by both spectral slope and Lempel–Ziv complexity. When derived from the full spectrum, slope and complexity capture redundant information and are influenced by oscillatory activity, particularly during sleep. In contrast, the narrowband slope (30–45 Hz) mainly indexes aperiodic brain activity, offering additional insights into underlying brain states. It provides a means to infer potential changes in the excitation/inhibition balance using only scalp electrodes, thus characterizing the spectral slope as a unique electrophysiological marker and distinguishing it from other measures such as spectral power. Moreover, recent evidence has shown that aperiodic activity on the scalp level can even offer insights into excitability changes on a cellular basis and reflects variations in pyramidal cell calcium activity (Lendner et al., 2023).

In the present study, the narrowband slope also proved to be the most powerful index of behavioral performance and was best suited to differentiate REM sleep from wakefulness and all other sleep stages without the additional use of EOG or EMG. Computing complexity in the narrowband range, however, did not yield any particularly meaningful results and should be undertaken with caution. Therefore, considering the faster computation time and the more diverse range of applications when using different frequency ranges as well as its correlation with task performance, the slope might be more practical and useful in most circumstances compared with complexity. Taken together, our study emphasizes the importance of considering the parametrization of brain activity into full, oscillatory, and aperiodic components to comprehensively understand the dynamics underlying passive and active brain states during sleep and wakefulness.

## Synthesis

Reviewing Editor: Alexander Soutschek, Ludwig-Maximilians-Universitat Munchen Decisions are customarily a result of the Reviewing Editor and the peer reviewers coming together and discussing their recommendations until a consensus is reached. When revisions are invited, a fact-based synthesis statement explaining their decision and outlining what is needed to prepare a revision will be listed below. The following reviewer(s) agreed to reveal their identity: Giulia Gaggioni, Vasil Kolev.

Reviewer 1

In this study, the authors computed spectral slope and LZ complexity in narrow and broadband frequency ranges across sleep stages and different cognitive tasks. The authors also analysed the relationship between spectral slope and LZ complexity, as well as the association of these parameters with task performance. I think this exploratory and extensive analysis is interesting for the scientific community and based on a unique and rich dataset (although not primarily designed for this specific study, as clarified by the authors). I compliment the authors for this original and well-written article.

My main questions would have been: 1. Why only men participants? ; 2. Why these tasks (rationale - e.g. is the Go/Nogo the best choice to infer attention level?)? ; 3. A possible lack of topographic information given the 11 electrodes. However, these points have already been addressed by the authors in the "Limitations" section.

Some additional minor points are:

p.5, line 94: for completeness, would it be possible to report the data of the entrance questionnaire or to refer to the paper with these pieces of information?

p.6, line 118: would it be possible to give more information about the different light conditions during the 90min break?

p.7, line 168: why did the authors choose to standardize the time in bed to 8 hours (11pm-7am)?

p.9, line 230: should maybe be the Figure 1 - 1 instead?

Method: was the computational time/effort comparable for both parameters?

p.12, line 294: Could you please clarify if REM was also significantly different from WAKE? Was WAKE included in the sleep classification comparison (Figure 4A)?

p.15, line 383: Figure 7 - 1: is "Slope (3-45Hz)" in the table correct?

p15 &16, section "The spectral slope as an electrophysiological marker of task performance". Lines 412 and 425: are these sentences fully supported by the values reported in figures 8 and 9? Similarly, p.17, line 451-452, is this assertion regarding the declarative memory task fully supported by the results?

p.21, section "The narrowband spectral slope as a unique marker of task performance":

-Q1: line 553: I am a bit confused here - would this also include the Go/Nogo third session?

-Q2: line 558: for completeness, should the authors also mention in this section effect size, confidence interval etc as complementary parameters of the p-value in larger scale studies?

Open question: given the protocol, just wondering if the authors investigated spectral slope/LZ complexity during Go/Nogo in function of time spent awake for broadband and narrowband?

Reviewer 2

The main aim of this paper is to determine how different brain states affect the spectral slope and Lempel-Ziv complexity measured in EEG data. Results are presented in excessive details which, in some parts of the paper, make it difficult to read. The whole paper is unduly long and needs shortening. The paper covers an interesting question and results are intriguing, but there are some problems which need further elaboration.

Questions

(1) The choice of broad-band and narrow-band EEG. Although the authors explain their arguments about fixing two frequency ranges, broad-band and narrow-band, it is still not clear why exactly these frequency ranges were chosen. Moreover, this choice may be problematic. Results of broad-band EEG analysis will be biased by the dominating EEG rhythm, which also may change during different brain states. This makes the outcome unstable and difficult to interpret. On other side, narrow-band EEG falls in the so-called gamma band, which may be severely influenced by EMG activity (as the authors correctly investigate) and produce unexpected variability reflected in both complexity and spectral slope. I have problems with comparing both frequency ranges in one and the same plot. Please argue explicitly the choice of bands and consider the confounding factors.

(2) The duration of brain states. As far as I understand, the authors have chosen 180 s long clean EEGs for analyses divided into 45 segments of 4 s. While for spontaneous EEG (respective sleep EEG) this is plausible, it may be problematic for investigations during task execution. 3 minute epochs during spontaneous states may show a relatively stationary process whereas during tasks, this may change drastically and the result will be contaminated. Here, analysis of event-related activity is missing to me. Please consider this possibility and/or discuss the issue.

(3) No comparison with analyses made with different methods. The paper presents only the results from the application of two analysis techniques, spectral slope and Lempel-Ziv complexity. No comparison is presented with other methods like spectral power, entropy, etc. This makes the study isolated from the huge bulk of existing analysis techniques in order to delineate specificity/advantages/contribution of the current approach. This issue should be at least tangentially considered.

(4) The relationship between the spectral slope and Lempel-Ziv complexity does not show meaningful results. This issue may be either discussed more deeply, or skipped.

(5) I am confused by the trivial conclusions. The reader will be eager to know what new neurophysiological mechanisms could be uncovered or highlighted by the application of these methods. All other statements in the Conclusion are already known.

(6) Legends and Figures.

- Unfortunately, I didn't succeed to open the figures in the *.eps files. Please convert them to pdf. I cannot understand the logic of dividing the figures into different parts - e.g., Fig. 1 A and B, Fig. 1-1 and 1-2, etc.

- The color titles in Figs. 2, 3, 5, 6 do not help to improve presentation. At the same time the x-axes are not labelled (Frequency [Hz]). What is [N] for the maps? Emphasize which frequency are the maps presenting?

- The reader automatically compares narrow-band data with the broad-band data in Fig. 4, but this is not meaningful. Probably, the two frequency plots should be separated?

Major

(1) The paper lacks consistency and compactness. At least half of the results (with Figures) can be transferred to Supplementary material.

(2) Discussion should be also shortened and focused to the main results.

(3) The contribution of the study should be clearly stated.

## Author Response

REBUTTAL - POINT BY POINT RESPONSE Please note that the original reviewer comments are given in BOLD type, while our response is given in blue. Modified passages in the manuscript, are written in red.

Reviewer #1:

In this study, the authors computed spectral slope and LZ complexity in narrow and broadband frequency ranges across sleep stages and different cognitive tasks. The authors also analysed the relationship between spectral slope and LZ complexity, as well as the association of these parameters with task performance. I think this exploratory and extensive analysis is interesting for the scientific community and based on a unique and rich dataset (although not primarily designed for this specific study, as clarified by the authors). I compliment the authors for this original and well-written article.

Thank you for appreciating our work and commending the manuscript. We highly appreciate your efforts regarding the review and try to address all remaining points below.

R1.1: My main questions would have been:

- Why only men participants? - Why these tasks (rationale - e.g., is the Go/Nogo the best choice to infer attention level?)? - A possible lack of topographic information given the 11 electrodes.

However, these points have already been addressed by the authors in the "Limitations" section.

As acknowledged above, we already addressed these questions within the limitations section of our original manuscript. Nonetheless, we would like to use the opportunity to revisit and elaborate on these issues again in more detail. We hope that we can offer a summarized and more concise overview of our rationale below:

The decision to exclude female participants stems from previously reported sex effects on sleep and light sensitivity, attributed to hormonal variations (Alonso et al., 2021; Chellappa et al., 2017; Plamberger et al., 2021). As the dataset comprises recordings of each participant over a time span of 14 days, attempting to control for hormonal variations by exclusively recording female participants in a specific menstrual phase (e.g., follicular) would have been extremely challenging: "Finally, we only recruited healthy biologically male adults in a restricted age range (18 - 25 years) to avoid potential sex differences and hormonal effects (Plamberger et al., 2021; Kozhemiako et al., 2022) and because controlling for hormonal variations by exclusively recording female participants in the follicular phase would have been extremely challenging with our study design. Therefore, it is unclear to what extent our results generalize to other populations." (lines 477 - 481) Given the complexity of our study design and the multitude of tasks, we opted for an attention task that could ideally measure various aspects in order to reduce the number of tasks included in the study. Consequently, we chose the Go/Nogo task over a classical psychomotor vigilance task to assess not only attention but also inhibition to some extent. Furthermore, previous studies demonstrated that the Go/Nogo task is sensitive to changes in general attention and cognitive control (e.g., Figueiro et al., 2016; Lesage et al., 2020; Terentjeviene et al., 2018) and measures not only error rates but also reaction times: "To assess objective levels of attention and inhibitory control, we implemented an auditory version of the Go/Nogo paradigm (Donders, 1969) via the Psychophysics Toolbox (PTB-3; Kleiner et al., 2007) in MATLAB (Release 2018b, The MathWorks Inc., Natick, MA). Due to the plentitude of tasks already included in the study design, we opted for an attention task that measures various aspects of attention and inhibitory control and therefore chose the Go/Nogo paradigm over a classical psychomotor vigilance task." (lines 123 - 128) Finally, we only utilized 11 scalp electrodes due to the need for long-term EEG data collection, spanning a whole night and several waking hours (approximately 14 - 16 hours per recording). Using electrode caps would have been more inconvenient for the participants and impractical without changing or reapplying them in between. Furthermore, manually applying more than 11 scalp electrodes would have been too time-consuming. Therefore, we opted for an 11-electrode setup (as done in other studies before, e.g., Hahn et al., 2022 or Webster and Ro, 2020), which still covered the main scalp areas (frontal, central, parietal, and occipital) but can be applied manually in a relatively short amount of time: "Despite using only 11 scalp electrodes and no high-density EEG caps due to the need for long-term EEG data collection (14 - 16 hours), we still robustly detected modulations by sleep stage and task engagement, thereby providing evidence for the practical usability of the slope and complexity as indices of different brain states. Nevertheless, research with high-density or intracranial EEG might further contribute to the understanding of which topographical areas are most influential in driving changes in slope or complexity across brain states." (lines 471 - 476) References:

Alonso A, Genzel L, Gomez A (2021) Sex and Menstrual Phase Influences on Sleep and Memory. Curr Sleep Medicine Rep 7:1-14.

Chellappa SL, Steiner R, Oelhafen P, Cajochen C (2017) Sex differences in light sensitivity impact on brightness perception, vigilant attention and sleep in humans. Sci Rep 7:14215.

Figueiro MG, Sahin L, Wood B, Plitnick B (2016) Light at Night and Measures of Alertness and Performance: Implications for Shift Workers. Biol Res Nurs 18:90-100.

Hahn MA, Bothe K, Heib D, Schabus M, Helfrich RF, Hoedlmoser K (2022) Slow oscillation-spindle coupling strength predicts real-life gross-motor learning in adolescents and adults. Elife 11.

Lesage E, Sutherland MT, Ross TJ, Salmeron BJ, Stein EA (2020) Nicotine dependence (trait) and acute nicotinic stimulation (state) modulate attention but not inhibitory control: converging fMRI evidence from Go-Nogo and Flanker tasks. Neuropsychopharmacology 45:857-865.

Plamberger CP, van Wijk HE, Kerschbaum H, Pletzer BA, Gruber G, Oberascher K, Dresler M, Hahn MA, Hoedlmoser K (2021) Impact of menstrual cycle phase and oral contraceptives on sleep and overnight memory consolidation. J Sleep Res 30:e13239.

Terentjeviene A, Maciuleviciene E, Vadopalas K, Mickeviciene D, Karanauskiene D, Valanciene D, Solianik R, Emeljanovas A, Kamandulis S, Skurvydas A (2018) Prefrontal Cortex Activity Predicts Mental Fatigue in Young and Elderly Men During a 2 h "Go/NoGo" Task. Front Neurosci 12:620.

Webster K, Ro T (2020) Visual Modulation of Resting State α Oscillations. eNeuro 7.

Minor Points:

R1.2: p.5, line 94: for completeness, would it be possible to report the data of the entrance questionnaire or to refer to the paper with these pieces of information? We apologize for not including this information before. A supplementary table that displays this data has now been included. We added the table as extended data to Figure 1 but also present it below for your convenience:

Figure 1 - 1. Entrance questionnaire results (mean and standard deviation; N = 28).

Note. Cut-Off values refer to recommended values for clinical or extreme populations.

R1.2: p.6, line 118: would it be possible to give more information about the different light conditions during the 90min break? We have now added further information regarding the light conditions to elaborate on this in more detail: "Afterwards, participants had a break of 1.5 hours in which they read standardized stories under different light conditions. These conditions consisted of either reading from a smartphone with or without a blue-light filter or from a printed book, leading to varying levels of short-wavelength light exposure with dim background room-lighting." (lines 114 - 117) Upon acceptance of the paper, this sentence will include a citation of one of our recent publications, in which these light conditions are described in even more detail. Unfortunately, it is not possible to include the citation at this point due to the double-blinded review policy. Specifically, light characteristics differed between the smartphone conditions in the following way: the display showed a melanopic radiance of 286.77mW*m-2*sr in the condition without a filter ("No Filter") and a melanopic radiance of 114.20mW*m-2*sr in the condition with a filter ("Filter"). The correlated colour temperature was at 8298K in the "No Filter" and at 3032K in the "Filter" condition (see Table R1). These light characteristics were measured using a spectrometer (height from floor to eye-level: 87cm, distance from eyes to stimulus: 37cm). In the "No Filter" and "Filter" conditions the stories were presented as e-book versions on the smartphone. In the "Book" condition, the stories were printed according to the format and text size of the e-book versions.

Table R1. Light source characteristics measured by JETI spectrometry.

Note. m-EDI = melanopic equivalent daylight illuminance. CCT = correlated color temperature.

All this information can also be found in the publication that is going to be cited for further information at the newly inserted passage above.

R1.3: p.7, line 168: why did the authors choose to standardize the time in bed to 8 hours (11pm-7am)? Our primary rationale for standardizing bedtimes was to mitigate strong interindividual differences arising from variations in sleep duration or sleep pressure. Choosing 8 hours for time in bed is common practice in many sleep laboratories and aligns with general recommendations for good sleep hygiene in (young) adults, acknowledging inherent interindividual differences: "Bedtime was scheduled for approximately 11:00 pm and participants were woken up 8 hours after lights out, thereby adhering to general sleep hygiene recommendations (Watson et al., 2015; Chaput et al., 2018)." (lines 107 - 109) The time window between 11pm - 7am was primarily selected for practical reasons, as it appeared to be well-accepted by the participants and could be easily adhered to throughout the study period. Additionally, later or earlier bedtimes would have posed challenges due to conflicting daytime recordings in our laboratory.

R1.4: p.9, line 230: should maybe be the Figure 1 - 1 instead? Thank you for spotting this mistake. We apologize for any confusion and have now renumbered the supplementary figures anyway since we added the results from the entrance questionnaire. Thus, this is now Figure 1 - 4 and has been updated accordingly, along with all other supplementary material related to Figure 1.

R1.5: Method: was the computational time/effort comparable for both parameters? To address your question, we re-ran both algorithms (slope vs. complexity calculation) on the same machine while storing the required computation times in MATLAB using the tic/toc functions. The slope calculation appears to be much quicker than the complexity calculation. In fact, calculating the spectral slope for all channels and all epochs of one task (45 epochs) from a single subject in 4 different frequency ranges is about 3.5 times faster than calculating LZ complexity with our implementations (73.16s vs. 264.50s).

This information has now also been included in the discussion section: "In contrast to complexity, the calculation of the slope was about 3.5 times more computationally efficient with our implementations, highlighting its practical applicability for closed loop and clinical settings." (lines 379 - 381) R1.6: p.12, line 294: Could you please clarify if REM was also significantly different from WAKE? Was WAKE included in the sleep classification comparison (Figure 4A)? We apologize for not making the differentiation between REM and WAKE clear enough. Yes, REM sleep was significantly different from wakefulness with all approaches but was best discernable when using the 30 - 45Hz slope (and least distinguishable with the 30 - 45Hz complexity). We added this information to the manuscript: "While REM sleep was significantly different from wakefulness in both frequency ranges for both parameters (all p < .025) it was best discernable with the narrowband slope." (lines 281 - 283) The sleep classification always involved all sleep stages (i.e., WAKE, N1, N2, N3 and REM). This can be seen implicitly by the chance level which is set at 20% in Figure 6 (which was Figure 4 in the original manuscript), but we made this clearer now by adding that information to the figure caption: "Figure 6. A: Comparison of the multi-class classification accuracies within 30 - 45Hz for the spectral slope and Lempel-Ziv complexity regarding sleep stages (WAKE, N1, N2, N3, REM) and tasks during wakefulness (resting eyes closed, resting eyes open, auditory Go/Nogo, encoding, retrieval). Sleep stages and tasks could be decoded more precisely with the spectral slope. Overall classification accuracy was significantly higher for tasks than for sleep stages. B: Comparison of the classification accuracies across tasks and sleep stages for slope and complexity within 1 - 45Hz. The slope only yielded better decoding performance during sleep, whereas task classification worked better when using Lempel-Ziv complexity, which is arguably due to the difference in complexity between the two resting conditions that is not present in the slope. The dotted red lines represent chance level (20%). [...]" R1.7: p.15, line 383: Figure 7 - 1: is "Slope (3-45Hz)" in the table correct? Thank you again for your attention to detail. Unfortunately, this was an error from our side and should be "Slope (1 - 45Hz)". We corrected the mistake, please note that this figure is now Figure 6 - 4 in the revised version of the manuscript.

R1.8.1: p15 &16, section "The spectral slope as an electrophysiological marker of task performance". Lines 412 and 425: are these sentences fully supported by the values reported in figures 8 and 9? Based on the line numbering of the original manuscript, you are referring to the following sentences:

1. "Only flatter slopes in the narrowband range (30 - 45Hz) were consistently related to higher task performance (see Figure 8)." In our opinion, this is supported by the data in Figure 8 as this figure shows that the narrowband slope correlated consistently positively with Go/Nogo task performance at all time points. Only in the third session, the overall correlation fails to reach statistical significance, but the general pattern is identical with the other time points, and most single electrodes still show a significant relationship. To avoid misleading communication, we chose not to use the word "significant" in the sentence you mentioned as, strictly speaking, this would only be true for three of the four time points. To make the sentence even more precise, we now rephrased it as follows: "Within the narrowband range, the slope was more consistently related to higher task performance than the complexity, reaching statistical significance at 3 out of 4 timepoints (cf., Figure 7)." (lines 341 - 342) 2. "Even though the overall pattern was similar to the Go/Nogo task, most correlation coefficients only showed a trend towards statistical significance (see Figure 9)." You are correct that this sentence could be understood in a misleading way to some extent. With a similar pattern to the Go/Nogo task, we refer to the observation that the correlation coefficients were also consistently positive. However, statistical significance was only reached at the delayed retrieval on electrodes C4 and P4, while all other correlations did not exceed statistical trends. Thus, we rephrased this sentence in the revised manuscript accordingly to communicate our findings more precisely: "Even though the slope was again consistently positively correlated with recall performance, only few correlations (C4 and P4 at the delayed retrieval) reached statistical significance (see Figure 8)." (lines 349 - 351) R1.8.2: Similarly, p.17, line 451-452, is this assertion regarding the declarative memory task fully supported by the results? Thank you for bringing to our attention that the claim might not be fully justified by the memory task data: "Critically, solely the narrowband spectral slope tracked task performance in an auditory attention task (Go/Nogo) as well as in a declarative memory task." We agree that, strictly statistically speaking, this can only be claimed for the Go/Nogo task since the correlations with the memory task were consistent but not significant at most electrodes. We apologize for simplifying that conclusion too much and rephrased that sentence so that it becomes clear that the claim regarding the memory task can only be tentatively: "However, (V) only the narrowband slope tracked task performance in an auditory attention task and trended towards significance regarding memory performance." (lines 371 - 373) R1.9: p.21, section "The narrowband spectral slope as a unique marker of task performance":

R1.9.1: line 553: I am a bit confused here - would this also include the Go/Nogo third session? You are referring to the sentence below: "When relating the spectral slope and Lempel-Ziv complexity to behavioral outcomes, we observed that only the narrowband slope within 30 - 45Hz was correlated consistently with attentional task performance in an auditory Go/Nogo task across all recordings per subject." We apologize again for this simplification of the results. You are correct that the third session does not show an overall significant correlation, which does not become clear in the sentence above. Again, we intended to simplify the result pattern since also the direction of the correlation was the same in the third session compared to all other sessions. To avoid any misunderstandings, we have adapted the sentence accordingly: "When relating slope and complexity to behavior, we observed that only the narrowband slope was consistently correlated with attentional task performance across almost all recordings per subject (with the exception of the third session, cf., Figure 7)." (lines 422 - 424) R1.9.2: line 558: for completeness, should the authors also mention in this section effect size, confidence interval etc as complementary parameters of the p-value in larger scale studies? Thank you for this suggestion, which refers to the following sentence: "In larger scale studies that rely on databases or in multicenter studies, which commonly have higher statistical power, however, the broadband slope and complexity were also significantly correlated with task performance." We agree that effect sizes and confidence intervals are important as additional information whenever possible. In theory, this would apply to our study too, but unfortunately, the non- and semi-parametrical multifactorial ANOVA-type tests that we conducted do not yet offer the option to calculate those metrics. Using classical ANOVAs would have been inappropriate in our case due to the violation of several assumptions (e.g., normally distributed data, outliers or homogenous variances). We have now updated the sentence above and mention the importance of those additional metrics: "However, in larger scale studies which offer more reliable effect sizes and confidence intervals, the broadband slope and complexity were also significantly correlated with task performance (Waschke et al., 2021; Mediano et al., 2021)." (lines 427 - 429) References:

Waschke L, Donoghue T, Fiedler L, Smith S, Garrett DD, Voytek B, Obleser J (2021) Modality-specific tracking of attention and sensory statistics in the human electrophysiological spectral exponent. Elife 10.

Mediano PAM, Ikkala A, Kievit RA, Jagannathan SR, Varley TF, Stamatakis EA, Bekinschtein TA, Bor D (2021) Fluctuations in Neural Complexity During Wakefulness Relate To Conscious Level and Cognition. bioRxiv. doi:10.1101/2021.09.23.461002.

R1.10: given the protocol, just wondering if the authors investigated spectral slope/LZ complexity during Go/Nogo in function of time spent awake for broadband and narrowband? Thank you for bringing up this topic. There has been recent research showing that a period of habitual sleep recalibrates the (broadband) spectral slope when comparing different tasks (resting, cognitive engagement and central fixation) from before and after sleep (Lendner et al., 2023). In that study, it had been reported that the slope steepens from before to after sleep. Thus, it is likely that we might observe a similar pattern with our Go/Nogo task data and could maybe also track some changes from the early evening until bedtime, which has not been covered in the cited paper above. Below, you can find an additional analysis addressing your question and comparing the broad- and narrowband slope and complexity during the Go/Nogo task over the different recordings (Figure R1). However, we decided to not include this finding in the revised manuscript in order to avoid introducing a new topic of discussion (i.e., the modulation of slope and complexity by sleep pressure and/or circadian variations). Additionally, given reviewer 2's request for a significantly shortened version, we prioritized brevity.

Figure R1. Modulation of spectral slope and LZ complexity by daytime (time spent awake). The spectral slope flattens in both frequency ranges over the course of the evening and steepens again after a night of sleep. LZ complexity is modulated similarly in the broadband frequency but appears to be unaffected in the narrowband range.

References:

Lendner JD, Niethard N, Mander BA, van Schalkwijk FJ, Schuh-Hofer S, Schmidt H, Knight RT, Born J, Walker MP, Lin JJ, Helfrich RF (2023) Human REM sleep recalibrates neural activity in support of memory formation. Sci Adv 9:eadj1895.- Reviewer #2:

The main aim of this paper is to determine how different brain states affect the spectral slope and Lempel-Ziv complexity measured in EEG data. Results are presented in excessive details which, in some parts of the paper, make it difficult to read. The whole paper is unduly long and needs shortening. The paper covers an interesting question and results are intriguing, but there are some problems which need further elaboration.

Thank you for taking the time to thoroughly review our manuscript and for providing us with valuable feedback. We appreciate your comments and agree that the paper is quite long due to the plentitude of analyses.

We now shortened the paper significantly (cf., our response to R2.7) in the results section from 2392 to 1513 words and in the discussion section from 2418 to 1971 words while still addressing the points that had been raised in the reviews (all changes are visible in the redline copy of the revised manuscript). Below, you can find a point-by-point response to all other specific points.

R2.1: The choice of broad-band and narrow-band EEG. Although the authors explain their arguments about fixing two frequency ranges, broad-band and narrow-band, it is still not clear why exactly these frequency ranges were chosen. Moreover, this choice may be problematic. Results of broad-band EEG analysis will be biased by the dominating EEG rhythm, which also may change during different brain states. This makes the outcome unstable and difficult to interpret. On other side, narrow-band EEG falls in the so-called gamma band, which may be severely influenced by EMG activity (as the authors correctly investigate) and produce unexpected variability reflected in both complexity and spectral slope. I have problems with comparing both frequency ranges in one and the same plot. Please argue explicitly the choice of bands and consider the confounding factors.

You are correct in stating that the choice of either a broad- or narrowband frequency range might be problematic since both have potential confounds. As you mentioned, the broadband might be influenced by oscillations and knees (i.e., bends in the power spectrum, which are a topic of its own and under current discussion in Donoghue et al., 2020 or Pani et al., 2022) and the narrowband is potentially influenced by muscular activity. Moreover, it generally remains unclear which frequency boundaries to choose for the calculation of narrowband aperiodic activity. Thus, this means that there is no ideal solution at the moment. Therefore, it is even more important to gain a better understanding of the pros and cons of different frequency-band choices (e.g., broadband vs. narrowband).

In summary, there is currently no consensus in the scientific community regarding a "best practice" approach in determining which frequency content and which frequency boundaries to use. Critically, there is not even a clear understanding of how different frequency ranges can affect the results. To address these issues, we wanted to employ and compare two very different but popular ranges (broadband: 1 - 45Hz and narrowband: 30 - 45Hz) that have been employed in several previous studies before (broadband: Ameen et al., 2023; Colombo et al., 2019; Helson et al., 2023; Jacob et al., 2021 vs. narrowband: Kozhemiako et al., 2022; Lendner et al., 2020; Lendner et al., 2022). Furthermore, exceeding 45Hz in EEG data is problematic due to line-noise at around 50Hz and was therefore not considered.

For the broadband range it would also be problematic to start from below 1Hz as this area typically exhibits another plateau that would require the fitting of an additional knee to not distort the actual slope values (He et al., 2010; Gerster et al., 2022). Regarding the narrowband range, setting the lower frequency limit to 30Hz was rooted in the original modeling work that demonstrated the capability of the spectral slope to track changes in excitation/inhibition balance (Gao et al., 2017). Critically, a large replicatory study with over 10.000 nights of sleep recordings confirmed the sensitivity of the 30 - 45Hz range for analyzing sleep EEG data (Kozhemiako et al., 2022), which makes this particular frequency band relatively well established and trustworthy. Additionally, Lendner et al. (2020) were able to show in a data-driven approach that the 25/30 - 45Hz range tracked the hypnogram the best: "(a) Fit center: Spearman rank correlation (blue, left panels) and Mutual Information (MI; green, right panels) between slope and hypnogram with different slope fits with center frequencies from 20 to 150 {plus minus} 10 Hz with SEM in intracranial data during sleep (n = 10). Upper left panel - R2 and lower left panel - p-value of Spearman rank correlation (blue). Upper right panel - MI and lower right panel - p-value of original MI observation tested against a surrogate MI distribution created by random block swapping (green). Red dotted lines for a p-value of 0.05. Black arrow indicates used center frequency of 40 Hz (30 to 50 Hz). (b) Fit length: Spearman rank correlation (blue, left panels) and Mutual Information (MI; green, right panels) with different slope fit lengths from 30 to 40 Hz up to 30 to 130 Hz (10 to 100 Hz fit length) with SEM in intracranial data during sleep (n = 10). Upper left panel - R2 and lower left panel - p-value of Spearman rank correlation (blue). Upper right panel - MI and lower right panel - p-value of original MI observation tested against a surrogate MI distribution created by random block swapping (green). Red dotted line for a p value of 0.05. Black arrow indicates used fit length for this study (20 Hz; 30 to 50 Hz). (c) Mixed (left) and fractal component (right) of power spectra in scalp EEG (n = 20) after IRASA. (d) Z-value of surrogate distribution (random block swapping) of Mutual Information (MI) between slope and hypnogram using the original (blue, 30 to 45 Hz) and different slope fits to fractal component (obtained by IRASA) in lower frequencies (scalp EEG, n = 20). Note that a z = 1.96 reflects an uncorrected two-tailed p-value of 0.05, while a z-score of >2.8 indicates a Bonferroni-corrected significant p-value (p<0.05/19 channels=0.0026)." (Figure 2 - figure supplement 5 from Lendner et al., 2020) Taken together, we adapted the following sections of the manuscript and now argue more explicitly why we chose the two frequency ranges:

Introduction (lines 57 - 68): "A big issue, however, that has hampered the assessment of and comparability between slope and complexity is the huge heterogeneity in frequency ranges used to calculate these measures. While there will be no optimal frequency range without potential confounds, it is important to compare different frequency ranges and calculation settings to gain a better understanding of how the choice of a certain frequency range affects the data. Particularly for the spectral slope, researchers have argued either in favor of broadband (Podvalny et al., 2015; Waschke et al., 2021; Karalunas et al., 2022) or more narrowband (Gao et al., 2017; Lendner et al., 2020) frequency ranges, commonly within 1 - 45/50Hz. While broadband ranges (e.g., 1 - 45Hz) encompass more of the total signal and result in better overall fits (Donoghue et al., 2020; Gerster et al., 2022), narrowband ranges (e.g., 30 - 45Hz) are less affected by slower oscillatory activity and reflect mostly aperiodic activity (Gao et al., 2017; Lendner et al., 2020). But also regarding complexity, recent evidence demonstrated that this metric is strongly affected by different frequency contents and might be mainly driven by lower frequencies (González et al., 2022)." Methods / Results (lines 253 - 266): "We calculated the spectral slope and Lempel-Ziv complexity for all sleep stages and tasks in a narrow- (30 - 45Hz) and broadband (1 - 45Hz) frequency range (cf., Figure 1B and C; Colombo et al., 2019; Lendner et al., 2020; Jacob et al., 2021; Lendner et al., 2022; Kozhemiako et al., 2022; Helson et al., 2023; Ameen et al., 2023). We set the upper frequency limit to 45Hz to avoid line-noise influences around 50Hz and the need for fitting a knee in higher frequencies. Likewise, we decided to set the lower frequency boundary for the broadband range at 1Hz since the area below often exhibits a plateau that would require fitting an additional knee or would otherwise also distort the data (He et al., 2010; Gerster et al., 2022). The 30Hz lower limit for the narrowband range was selected based on original modeling work for the spectral slope, demonstrating that the 30 - 45Hz range is capable of tracking changes in the brain's excitation / inhibition balance (Gao et al., 2017). Furthermore, a large replication study with more than 10.000 polysomnography recordings confirmed that the 30 - 45Hz range tracks the hypnogram accurately (Kozhemiako et al., 2022), a finding that has also been supported by others who used a data-driven fitting algorithm to compare different frequency ranges for slope estimation (Lendner et al., 2020; Lendner et al., 2022)." As you mentioned, we also conducted additional analyses in which we partialled out the EMG activity from the EEG to address your concern around the influence of the EMG. For this, we calculated the 30 - 45Hz slope and complexity from all scalp-EEG channels and from the EMG for each epoch of all sleep stages and tasks. We then correlated the epoch-wise slope and complexity values from all scalp channels with the sleep stages and tasks and ran partial correlations accounting for the slope and complexity from the EMG. Lastly, we assessed the inter-correlation between the EEG and EMG slope and complexity.

We were able to show that the 30 - 45Hz slope was not strongly contaminated by EMG activity (cf., Figure 2 - 1) since it was negatively correlated with sleep stage (i.e., steeper slopes from WAKE to REM) and because this correlation remained unaffected by partialling out the EMG. During wakefulness, more task engagement was associated with flatter EEG narrowband slopes, independently from the EMG slope.

Figure 2 - 1. Control analyses including the narrowband spectral slope from the EMG. A: The negative correlations between EEG slope and sleep stage do not change when partialling out the EMG slope. B: While the average EEG slope is negatively correlated with sleep stage, the EMG slope is even slightly positively correlated with sleep stage and significantly different from the EEG slope correlation. C: The positive correlations between EEG slope and the cognitive tasks (ordered ascendingly regarding their slope) are not diminished when controlling for the EMG. D: While the correlation between the EMG slope and the tasks is slightly higher than between the EEG slope and the tasks, partialling out the EMG from the EEG slope does not significantly reduce the correlation. E & F: Differential modulation of the EEG & EMG slopes across sleep stages and tasks.

While the general results of the narrowband complexity were not as straightforward per se, also the narrowband complexity was relatively unaffected by the EMG as removing the EMG from the EEG complexity did not change the correlations between complexity values and sleep stage or task engagement (cf., Figure 2 - 2).

Figure 2 - 2. Control analyses including the narrowband Lempel-Ziv complexity (LZC) from the EMG. A: The positive correlations between EEG complexity and sleep stage do not change when partialling out the EMG complexity. B: While both, the average EEG and EMG complexity are positively correlated with sleep stage, the partial correlation controlling for EMG complexity does not shrink substantially. C: The negative correlations between EEG complexity and the cognitive tasks are not changed substantially by partialling out the EMG. D: Both, the average EEG and EMG complexity are negatively correlated with the tasks during wakefulness but the partial correlation between EEG complexity and the tasks controlled for the EMG is not significantly smaller. E & F: Differential modulation of the EEG & EMG complexity across sleep stages and tasks.

Furthermore, there is research demonstrating that the 30 - 45Hz range is even more sensitive to brain state alterations during sleep or under anesthesia in intracranial data (Gao et al., 2017; Lendner et al., 2020), which is free of muscular artifacts. Thus, we believe that the 30 - 45Hz range carries meaningful information independent from muscular activity.

Regarding the comparison of both frequency ranges in one and the same plot, we address this in more detail in R2.6.3 and tried to separate these comparisons.

References:

Ameen MS, Petzka M, Peigneux P, Hoedlmoser K (2023) Post-training sleep modulates motor adaptation and task-related beta oscillations. J Sleep Res:e14082.

Colombo MA, Napolitani M, Boly M, Gosseries O, Casarotto S, Rosanova M, Brichant J-F, Boveroux P, Rex S, Laureys S, Massimini M, Chieregato A, Sarasso S (2019) The spectral exponent of the resting EEG indexes the presence of consciousness during unresponsiveness induced by propofol, xenon, and ketamine. Neuroimage 189:631-644.

Donoghue T, Haller M, Peterson EJ, Varma P, Sebastian P, Gao R, Noto T, Lara AH, Wallis JD, Knight RT, Shestyuk A, Voytek B (2020) Parameterizing neural power spectra into periodic and aperiodic components. Nat Neurosci 23:1655-1665.

Gao R, Peterson EJ, Voytek B (2017) Inferring synaptic excitation/inhibition balance from field potentials. Neuroimage 158:70-78.

Gerster M, Waterstraat G, Litvak V, Lehnertz K, Schnitzler A, Florin E, Curio G, Nikulin V (2022) Separating Neural Oscillations from Aperiodic 1/f Activity: Challenges and Recommendations. Neuroinformatics. doi:10.1007/s12021-022-09581-8.

He BJ, Zempel JM, Snyder AZ, Raichle ME (2010) The temporal structures and functional significance of scale-free brain activity. Neuron 66:353-369.

Helson P, Lundqvist D, Svenningsson P, Vinding MC, Kumar A (2023) Cortex-wide topography of 1/f-exponent in Parkinson's disease. NPJ Parkinsons Dis 9:109.

Jacob MS, Roach BJ, Sargent KS, Mathalon DH, Ford JM (2021) Aperiodic measures of neural excitability are associated with anticorrelated hemodynamic networks at rest: A combined EEG-fMRI study. Neuroimage 245:118705.

Kozhemiako N, Mylonas D, Pan JQ, Prerau MJ, Redline S, Purcell SM (2022) Sources of variation in the spectral slope of the sleep EEG. eNeuro 9.

Lendner JD, Helfrich RF, Mander BA, Romundstad L, Lin JJ, Walker MP, Larsson PG, Knight RT (2020) An electrophysiological marker of arousal level in humans. Elife 9.

Lendner JD, Mander BA, Schuh-Hofer S, Schmidt H, Knight RT, Walker MP, Lin JJ, Helfrich RF (2022) Human REM sleep controls neural excitability in support of memory formation. bioRxiv. doi:10.1101/2022.05.13.491801.

Pani SM, Saba L, Fraschini M (2022) Clinical applications of EEG power spectra aperiodic component analysis: A mini-review. Clin Neurophysiol 143:1-13.

R2.2: The duration of brain states. As far as I understand, the authors have chosen 180 s long clean EEGs for analyses divided into 45 segments of 4 s. While for spontaneous EEG (respective sleep EEG) this is plausible, it may be problematic for investigations during task execution. 3 minute epochs during spontaneous states may show a relatively stationary process whereas during tasks, this may change drastically and the result will be contaminated. Here, analysis of event-related activity is missing to me. Please consider this possibility and/or discuss the issue.

You are correct that we always used 4s segments for all tasks and sleep stages. However, the restriction to 45 epochs was only implemented for the multivariate pattern analyses since a comparable number of epochs is required for those to avoid significant biases in classification performance. For all other comparisons and for the correlations with behavior, we used as many epochs as we were able to obtain per task or sleep stage in order to improve data quality and reliability (cf., supplementary table in Figure 1 - 3).

Figure 1 - 3. Mean number of clean epochs (min, max) for all tasks and sleep stages per experimental condition (i.e., different lab-visits). For the wakefulness recordings, the data is averaged over the multiple measurements per lab-visit and the encoding session has been pooled over both runs per visit (N = 28).

The rationale for using 4s segments was that this enabled the best tradeoff between a sufficient number of epochs even for the shortest tasks (3min resting sessions, i.e., 45 epochs) while maintaining an adequate frequency resolution. We tried to avoid varying epoch lengths between tasks and sleep stages as this could especially influence the estimates of LZ complexity (cf., Figure R2) and might render different tasks incomparable.

Figure R2. Effect of different epoch-lengths (1s vs. 4s) for the Go/Nogo task on the spectral slope (A) and Lempel-Ziv complexity (B) calculations. Data is presented averaged over all electrodes and repeated measurements per subject. Lempel-Ziv complexity increases significantly with shorter computation windows in both frequency ranges (p < .001).

Nevertheless, we agree with you that during active task execution, the slope and complexity might not be stationary or comparable throughout the task and could vary between periods with or without events. We calculated the slope and complexity over the whole duration of a task or sleep stage (similar to the rationale of Mediano et al., 2021) because we aimed to compare overall task or sleep stage related differences and not stimulus-based changes.

However, we have now conducted an additional analysis to better address your question and compared the slope and complexity during the Go/Nogo task between interstimulus intervals (ISI) and GO or NOGO intervals. For these analyses, we used 1s segments that were either extracted from intervals between stimulus presentation and therefore were not affected by external stimulation (-1250ms to -250ms relative to a GO or NOGO signal) or segments that were extracted around a GO or NOGO signal (-250ms to 750ms relative to the stimulus). We compared these slope and complexity estimates against the original values obtained from the continuous 4s epochs across the whole task (cf., Figure 5 - 3 below). As shown above, the choice of different epoch lengths strongly affected the complexity values, but neither the complexity nor the slope showed strong variations between the different types of epochs (GO, NOGO, ISI or ALL). While this might not be too surprising for the narrowband as this frequency range is hardly affected by resulting ERPs, one might have expected a stronger effect on the broadband range. On a descriptive basis there is indeed more variation between the different epoch types in the broadband frequency range that was, however, too weak to yield statistical significance. Thus, in the future it might indeed be promising to focus in more detail on potential influences of slope and/or complexity due to stimulus-dependent changes during tasks.

This additional analysis has been added as a supplementary figure to the revised manuscript:

Figure 5 - 3. Effect of choosing 1s epochs around a stimulus (GO or NOGO) or 1s epochs during an interstimulus interval (ISI) or 4s epochs across the whole task. While the general epoch-length has a strong impact on the complexity estimates (C - D), neither the slope nor the complexity is strongly affected by different epoch-types (A - D).

Results (lines 317 - 319; GNG task = Go/Nogo task): "Further, we tested in the GNG task whether it would have made a difference if we focused only on epochs with or without stimulus presentation and did not detect strong differences between different epoch types (cf., Figure 5 - 3)." References:

Mediano PAM, Ikkala A, Kievit RA, Jagannathan SR, Varley TF, Stamatakis EA, Bekinschtein TA, Bor D (2021) Fluctuations in Neural Complexity During Wakefulness Relate To Conscious Level and Cognition. bioRxiv. doi:10.1101/2021.09.23.461002.

R2.3: No comparison with analyses made with different methods. The paper presents only the results from the application of two analysis techniques, spectral slope and Lempel-Ziv complexity. No comparison is presented with other methods like spectral power, entropy, etc. This makes the study isolated from the huge bulk of existing analysis techniques in order to delineate specificity/advantages/contribution of the current approach. This issue should be at least tangentially considered.

We agree about the abundance of different analysis techniques that could potentially serve similarly well in differentiating brain states during sleep or wakefulness. For instance, spectral power (as you mentioned) is of course widely recognized for its modulation by sleep in lower frequency ranges and in response to task engagement in higher frequencies. However, a comprehensive comparison of the spectral slope and Lempel-Ziv with other methods, such as variations in different power bands, entropy measures, or other derivates of the EEG would exceed the scope of our paper. This is primarily due to the need for an extensive discussion and comparison of these different approaches in terms of both their intrinsic qualities and how they should be implemented and finetuned. Additionally, our aim was to specifically contrast spectral slope and Lempel-Ziv complexity against another, given their common usage and the existing literature suggesting their similarity in response to brain state changes during sleep or wakefulness (Zhang et al., 2001; Ferenets et al., 2007; Andrillon et al., 2016; Schartner et al., 2017; Ma et al., 2018; Colombo et al., 2019; Miskovic et al., 2019; Waschke et al., 2021).

While we acknowledge the absence of direct comparisons with other methods in the manuscript, recognizing the importance of such comparisons for future debates about parameter selection, we have now added a dedicated paragraph to the discussion section.

Discussion / Limitations (lines 452 - 462): "In this study, we focused exclusively on two specific EEG derivates: spectral slope and Lempel-Ziv complexity. Consequently, we did not compare these measures against other established analytical techniques such as spectral power, entropy, or network analyses (e.g., coherence). However, regarding spectral power, previous research demonstrated that the spectral slope is superior in discriminating REM sleep from wakefulness compared to slow oscillation or gamma power, and performs similarly to beta power (Lendner et al., 2020; Kozhemiako et al., 2022). Additionally, Biggs et al. (2022) showed that Lempel-Ziv complexity also performed similarly well in comparison to alpha power and outperformed permutation entropy in capturing age-related changes in brain activity induced by anesthesia. However, comparisons with other biomarkers, such as heart rate variability or blood pressure (Radha et al., 2019; Mitsukura et al., 2020; Kuula and Pesonen, 2021), as well as specific benchmark tests involving both spectral slope and Lempel-Ziv complexity, are lacking and merit exploration in future large datasets." References:

Andrillon T, Poulsen AT, Hansen LK, Léger D, Kouider S (2016) Neural Markers of Responsiveness to the Environment in Human Sleep. The Journal of neuroscience 36:6583-6596.

Colombo MA, Napolitani M, Boly M, Gosseries O, Casarotto S, Rosanova M, Brichant J-F, Boveroux P, Rex S, Laureys S, Massimini M, Chieregato A, Sarasso S (2019) The spectral exponent of the resting EEG indexes the presence of consciousness during unresponsiveness induced by propofol, xenon, and ketamine. Neuroimage 189:631-644.

Ferenets R, Vanluchene A, Lipping T, Heyse B, Struys MMRF (2007) Behavior of entropy/complexity measures of the electroencephalogram during propofol-induced sedation: dose-dependent effects of remifentanil. Anesthesiology 106:696-706.

Ma Y, Shi W, Peng C-K, Yang AC (2018) Nonlinear dynamical analysis of sleep electroencephalography using fractal and entropy approaches. Sleep Med Rev 37:85-93.

Miskovic V, MacDonald KJ, Rhodes LJ, Cote KA (2019) Changes in EEG multiscale entropy and power-law frequency scaling during the human sleep cycle. Hum Brain Mapp 40:538-551.

Schartner M, Pigorini A, Gibbs SA, Arnulfo G, Sarasso S, Barnett L, Nobili L, Massimini M, Seth A, Barrett AB (2017) Global and local complexity of intracranial EEG decreases during NREM sleep. Neurosci Conscious 2017:1-12.

Waschke L, Donoghue T, Fiedler L, Smith S, Garrett DD, Voytek B, Obleser J (2021) Modality-specific tracking of attention and sensory statistics in the human electrophysiological spectral exponent. Elife 10.

Zhang XS, Roy RJ, Jensen EW (2001) EEG complexity as a measure of depth of anesthesia for patients. IEEE Trans Biomed Eng 48:1424-1433.

R2.4: The relationship between the spectral slope and Lempel-Ziv complexity does not show meaningful results. This issue may be either discussed more deeply or skipped.

After considering the overall length of the paper and the focus on the difference between narrow- and broadband ranges as well as the applicability of the two parameters for assessing differences between sleep stages, tasks as well as their relationship with task performance, we agree with you and decided to move the correlations between slope and complexity entirely to the supplementary material. Thus, we now only touch on the observation that the two parameters might share some properties and behave similarly, at least in a broadband frequency range. We hope that this also helps with making the paper more concise and streamlined.

Consequently, the chapter "Relationship between the spectral slope and Lempel-Ziv complexity" has been dropped completely from the results section and some paragraphs that were elaborating on this in the introduction and discussion were removed and/or restructured. The following paragraph has been added to the results section to refer to the supplementary material regarding the relationship between the two parameters: "An overview of the pairwise classification accuracies for all sleep stages and task pairings is presented in Figures 6 - 1 & 6 - 2. Correlations between slope and complexity as well as analyses of the robustness across repeated measurements are presented in Figures 6 - 3, 6 - 4 and 6 - 5." (lines 330 - 333) R2.5.: I am confused by the trivial conclusions. The reader will be eager to know what new neurophysiological mechanisms could be uncovered or highlighted by the application of these methods. All other statements in the Conclusion are already known.

Thank you for drawing our attention to the fact that the paper would benefit from highlighting the specific contributions of our study. In the revised version, we stress that analyzing aperiodic brain activity, especially via the spectral slope, allows to infer changes in the brain's excitability without the use of invasive methods, which is not feasible with other electrophysiological measures (e.g., spectral power). Specifically, aperiodic electrophysiological activity has very recently been shown to reflect pyramidal cell activity in sleep and to modulate slow oscillatory activity (Lendner et al., 2023). Thus, especially the spectral slope might constitute an easily obtainable EEG marker that can provide insights into cellular events - a unique capability that provides additional value in comparison to other scalp-EEG derivates.

Furthermore, we tried to provide some guidance regarding which parameter might be better suited for future analyses and what should be considered regarding the choice of different frequency ranges. Overall, we conclude that in most circumstances the spectral slope might be more useful and practical than Lempel-Ziv complexity as it is much quicker and easier to calculate and conveys mostly redundant information when using the full-spectrum but provides additional value in a restricted frequency range (30 - 45Hz). Thus, the conclusion has been rewritten as follows:

Discussion / Conclusions (lines 483 - 504): "Our results demonstrate that EEG spectral slope and Lempel-Ziv complexity are powerful indices of brain states during sleep and wakefulness. We present robust evidence from multiple recordings of three within-subject measurements, revealing that sleep stages and various cognitive tasks are reliably indexed by both spectral slope and Lempel-Ziv complexity. When derived from the full spectrum, slope and complexity capture redundant information and are influenced by oscillatory activity, particularly during sleep. In contrast, the narrowband slope (30 - 45Hz) mainly indexes aperiodic brain activity, offering additional insights into underlying brain states. It provides a means to infer potential changes in the excitation/inhibition balance using only scalp electrodes, thus characterizing the spectral slope as a unique electrophysiological marker and distinguishing it from other measures such as spectral power. Moreover, recent evidence has shown that aperiodic activity on the scalp level can even offer insights into excitability changes on a cellular basis and reflects variations in pyramidal cell calcium activity (Lendner et al., 2023).

In the present study, the narrowband slope also proved to be the most powerful index of behavioral performance and was best suited to differentiate REM sleep from wakefulness and all other sleep stages without the additional use of EOG or EMG. Computing complexity in the narrowband range, however, did not yield any particularly meaningful results and should be undertaken with caution. Therefore, considering the faster computation time and the more diverse range of applications when using different frequency ranges as well as its correlation with task performance, the slope might be more practical and useful in most circumstances compared to complexity. Taken together, our study emphasizes the importance of considering the parametrization of brain activity into full, oscillatory and aperiodic components to comprehensively understand the dynamics underlying passive and active brain states during sleep and wakefulness." References:

Lendner JD, Niethard N, Mander BA, van Schalkwijk FJ, Schuh-Hofer S, Schmidt H, Knight RT, Born J, Walker MP, Lin JJ, Helfrich RF (2023) Human REM sleep recalibrates neural activity in support of memory formation. Sci Adv 9:eadj1895.

R2.6: Legends and Figures.

R2.6.1: Unfortunately, I didn't succeed to open the figures in the *.eps files. Please convert them to pdf. I cannot understand the logic of dividing the figures into different parts - e.g., Fig. 1 A and B, Fig. 1-1 and 1-2, etc.

We apologize for any inconvenience caused by the figure format. Due to the journal regulations, acceptable formats for figures are only *.eps or *.tiff files. Consequently, we have now converted all figures to *.tiff and hope that this resolves the issues you encountered.

The rationale behind dividing figures into different parts was to avoid overloading single figures, especially considering the complexity of some. Our intention was to present certain content as supplementary information and to provide structure to complex figures by organizing them into sections (e.g., A, B, C) rather than creating entirely new figures. This approach was taken to prevent a significant increase in the number of display items. Thus, if letters are used in reference to a figure, then this means that this only refers to a section of the figure (e.g., to the section A of Figure 1 that has the panels A, B and C).

According to the journal guidelines, supplementary information (i.e., extended data) is only allowed in the form of tables or figures relating to main figures. Thus, Figure 1 - 1 and Figure 1 - 2 would refer to supplementary figures that are still related to the overall topic of Figure 1 but are, in our opinion, not necessary to be included in the main manuscript. We hope this explains our decision to name the different figures and parts accordingly.

R2.6.2: The color titles in Figs. 2, 3, 5, 6 do not help to improve presentation. At the same time the x-axes are not labelled (Frequency [Hz]). What is [N] for the maps? Emphasize which frequency are the maps presenting? Thank you for your suggestions to improve figure clarity and understandability. We changed the colored titles back to black and added x-axes labels accordingly in Figures 2, 3, 5 and 6 as well as in the corresponding supplementary figures. The sample size for each figure is always mentioned in the figure caption and was N = 27 in Figures 2 & 3 and N = 28 in Figures 5 & 6. The [N] on the maps was intended to be a "z" that had been rotated by 90{degree sign} (the topoplots represent z-values of the slope or complexity computed from the grand average across all sleep stages or tasks to better indicate topographical deviations). In order to avoid such misunderstandings for future readers, we now wrote the "z" horizontally [z] instead of vertically.

As an example, please find Figure 2 and its caption below:

Figure 2. Spectral slope (green, A) and Lempel-Ziv (LZ) complexity (purple, B) from 30 - 45Hz across sleep, averaged over all lab-sessions per subject. Center figures show the data averaged over all electrodes and topographical maps are provided below (color-coding refers to z-values of slope or complexity computed from the grand average across all sleep stages). In A, the log-log power spectra are provided for each sleep stage to illustrate the slope changes across different sleep stages. Classification accuracies are shown on the right-hand side. A: The spectral slope decreases from wakefulness across all sleep stages to REM sleep with a small temporary increase during N3 sleep. B: Lempel-Ziv complexity increases from shallow N1 to light N2 sleep and is in general less modulated by sleep stage than the spectral slope. EMG activity did not confound the modulation of the spectral slope and Lempel-Ziv complexity during sleep (see Figure 2 - 1) or wakefulness (see Figure 2 - 2). ***: p < .001, **: p {less than or equal to} .010, *: p {less than or equal to} .050, n.s.: p > .050; all p-values are adjusted for multiple comparisons; error-bars represent 95% confidence intervals (N = 27).

R2.6.3: The reader automatically compares narrow-band data with the broad-band data in Fig. 4, but this is not meaningful. Probably, the two frequency plots should be separated? We thank you for this suggestion. We now changed this Figure and A and B are not separated into "Sleep Classification" and "Wake Classification" but into "30 - 45Hz" and "1 - 45Hz". We agree that this increases the clarity of the figure and is more fitting to the overall storyline (please note that due to some re-organization of the manuscript, this is now Figure 6). Thus, the reader now compares sleep and wake classification within the same frequency band:

Figure 6. A: Comparison of the multi-class classification accuracies within 30 - 45Hz (see Figure 6 - 1 for all pairwise classification accuracies) for the spectral slope and Lempel-Ziv complexity regarding sleep stages (WAKE, N1, N2, N3, REM) and tasks during wakefulness (resting eyes closed, resting eyes open, auditory Go/Nogo, encoding, retrieval). Sleep stages and tasks could be decoded more precisely with the spectral slope. Overall classification accuracy was significantly higher for tasks than for sleep stages. B: Comparison of the classification accuracies across tasks and sleep stages for slope and complexity within 1 - 45Hz (see Figure 6 - 2 for the pairwise classifications). The slope only yielded better decoding performance during sleep, whereas task classification worked better when using Lempel-Ziv complexity, which is arguably due to the difference in complexity between the two resting conditions that is not present in the slope. The dotted red lines represent chance level (20%). The correlation between slope and complexity within 30 - 45Hz and 1 - 45Hz is presented in Figure 6 - 3. Figure 6 - 4 and Figure 6 - 5 demonstrate the robustness of the slope and complexity values across lab-visits and the correlation across frequency ranges. ***: p < .001, n.s.: p > .050 (N = 28).

Major R2.7: The paper lacks consistency and compactness. At least half of the results (with Figures) can be transferred to Supplementary material.

Discussion should be also shortened and focused to the main results.

Unfortunately, the transfer of manuscript sections to supplementary material is restricted by journal guidelines, which only permit figures or tables in supplementary material as extended data. Hence, relocating text to supplementary material is very limited and only possible in the form of figure captions. However, we still shortened the manuscript significantly by:

1. Rephrasing and shortening some methodological descriptions.

2. Rewriting the results section in a less narrative style and omitting detailed reports of pairwise comparisons that are already highlighted and emphasized in figures.

3. Removing the results section about the correlation between slope and complexity (cf., R2.4) and relocating the corresponding figures to the extended data.

4. Shortening the discussion section by providing more concise elaborations, eliminating points not directly related to main results, and omitting the discussion of the relationship between slope and complexity.

Multiple paragraphs have been rephrased and, in parts, some sections were expanded due to the issues that were raised by the reviews. We believe that the revised manuscript now conveys a more consistent and easy-to-follow storyline.

R2.8: The contribution of the study should be clearly stated.

Thank you for highlighting again the need for a clearer conclusion that enables readers to see the significance and contribution of our manuscript more easily. As already described in detail in response to R2.5, we tried to put this more clearly by addressing potential underlying neurophysiological mechanisms, highlighting the practical value of analyzing aperiodic activity and advocating in favor of one of the two parameters based on our results and the practical usability.
