# Supplementary material for: Spectral Slope and Lempel–Ziv Complexity as Robust Markers of Brain States during Sleep and Wakefulness
Source: eNeuro. 2024 Mar 25;11(3):ENEURO.0259-23.2024. doi: 10.1523/ENEURO.0259-23.2024 (PMC10978822; doi:10.1523/ENEURO.0259-23.2024)
Supplement: Figure 1-1. — Entrance questionnaire results (mean and standard deviation; N = 28). Download Figure 1-1, DOCX file. [file eneuro-11-ENEURO.0259-23.2024-s002.docx]

**Figure 1 – 1. Entrance questionnaire results (mean and standard deviation; *N* = 28).**

| Questionnaire | Mean (SD) | Cut-Off |
| --- | --- | --- |
| Pittsburgh Sleep Quality Index | 3.36 (2.11) | ≥ 10 |
| State Trait Anxiety Inventory: Trait | 33.39 (8.39) | ≥ 45 |
| Social Interaction Anxiety Scale | 23.28 (15.75) | ≥ 30 |
| Beck-Depression-Inventory | 3.96 (4.44) | ≥ 18 |
| Perceived Stress Scale | 10.36 (5.14) | ≥ 27 |
| Morning-Eveningness Questionnaire | 53.96 (8.58) | ≤ 30 or ≥ 70 |

**Note.** Cut-Off values refer to recommended values for clinical or extreme populations.
