# Supplementary material for: Spectral Slope and Lempel–Ziv Complexity as Robust Markers of Brain States during Sleep and Wakefulness
Source: eNeuro. 2024 Mar 25;11(3):ENEURO.0259-23.2024. doi: 10.1523/ENEURO.0259-23.2024 (PMC10978822; doi:10.1523/ENEURO.0259-23.2024)
Supplement: Figure 1-2. — Whole night sleep architecture for all lab visits (median and interquartile range; N = 28). Download Figure 1-2, DOCX file. [file eneuro-11-ENEURO.0259-23.2024-s003.docx]

**Figure 1 – 2. Whole night sleep architecture.**

|  | Adapt | Exp. Recording #1 | Exp. Recording #2 | Exp. Recording #3 |
| --- | --- | --- | --- | --- |
| TIB (min) | 480.75 (1.00) | 480.50 (0.13) | 480.50 (0.50) | 480.50 (0.50) |
| TST (min) | 461 (23.88) | 466 (27.63) | 464.25 (18.75) | 468.50 (15.75) |
| SEFF (%) | 95.85 (4.99) | 97.14 (5.18) | 96.83 (4.08) | 97.50 (2.91) |
| SOL N2 (min) | 17.50 (12.25) | 12 (6.88) | 11.25 (9.38) | 11 (6.82) |
| N1 (%) | 15.45 (6.82) | 12.77 (7.00) | 10.40 (6.14) | 10.59 (6.14) |
| N2 (%) | 40.69 (12.27) | 38.32 (8.32) | 39.38 (8.07) | 39.01 (7.12) |
| N3 (%) | 27.43 (10.45) | 28.13 (11.64) | 29.28 (6.72) | 29.32 (8.69) |
| REM (%) | 15.46 (6.45) | 19.58 (7.27) | 20.43 (7.88) | 20.10 (5.58) |
| WASO (min) | 11.50 (25.75) | 9 (13.5) | 10.25 (11.88) | 7.75 (10.25) |

**Note.** TIB = Time in bed, TST = Total sleep time, SEFF = Sleep efficiency, SOL N2 = Sleep onset latency to N2, WASO = Wake time after sleep onset.
