# Supplementary material for: Spectral Slope and Lempel–Ziv Complexity as Robust Markers of Brain States during Sleep and Wakefulness
Source: eNeuro. 2024 Mar 25;11(3):ENEURO.0259-23.2024. doi: 10.1523/ENEURO.0259-23.2024 (PMC10978822; doi:10.1523/ENEURO.0259-23.2024)
Supplement: Figure 1-3. — Mean number of clean epochs (min, max) for all tasks and sleep stages per experimental condition (i.e., different lab-visits). For the wakefulness recordings, the data is averaged over the multiple measurements per lab-visit and the encoding session has been pooled over both runs per visit (N = 28). Download Figure 1-3, DOCX file. [file eneuro-11-ENEURO.0259-23.2024-s004.docx]

**Figure 1 – 3.** **Mean number of clean epochs (min, max) for all tasks and sleep stages per experimental condition (i.e., different lab-visits). For the wakefulness recordings, the data is averaged over the multiple measurements per lab-visit and the encoding session has been pooled over both runs per visit (*N* = 28).**

| **A) Epochs for the multivariate pattern analyses (MVPA)** | | | |
| --- | --- | --- | --- |
| Task / Sleep-Stage | Exp. Recording #1 | Exp. Recording #2 | Exp. Recording #3 |
| Resting eyes closed | 42 (36, 45) | 41 (27, 45) | 42 (26, 45) |
| Resting eyes open | 41 (34, 45) | 39 (16, 45) | 41 (30, 45) |
| Go/Nogo task | 45 (40, 45) | 45 (39, 45) | 45 (45, 45) |
| Encoding | 46 (46, 46) | 46 (46, 46) | 46 (46, 46) |
| Retrieval | 43 (19, 45) | 43 (24, 45) | 43 (20, 45) |
| Wake (stage) | 45 (45, 45) | 45 (45, 45) | 45 (44, 45) |
| NREM1-3 & REM | 45 (45, 45) | 45 (45, 45) | 45 (45, 45) |
| **B) Epochs for all other analyses** | | | |
| Task / Sleep-Stage | Exp. Recording #1 | Exp. Recording #2 | Exp. Recording #3 |
| Resting eyes closed | 43 (36, 53) | 42 (27, 52) | 43 (26, 48) |
| Resting eyes open | 42 (34, 48) | 39 (16, 46) | 41 (30, 47) |
| Go/Nogo task | 143 (55, 165) | 136 (91, 165) | 138 (91, 161) |
| Encoding | 307 (65, 400) | 302 (168, 402) | 324 (208, 409) |
| Retrieval | 96 (19, 191) | 90 (24, 145) | 96 (20, 171) |
| Wake (stage) | 353 (47, 2099) | 301 (71, 1735) | 212 (44, 616) |
| NREM1 | 843 (395, 1748) | 848 (335, 2526) | 821 (415, 1453) |
| NREM2 | 2614 (1212, 3387) | 2585 (1244, 3339) | 2688 (1893, 3417) |
| NREM3 | 1916 (1109, 3220) | 1939 (1032, 3500) | 1966 (1233, 2940) |
| REM | 1224 (395, 1981) | 1312 (523, 1918) | 1327 (540, 1946) |
