# Supplementary figures and images for: Spectral Slope and Lempel–Ziv Complexity as Robust Markers of Brain States during Sleep and Wakefulness
Source: eNeuro. 2024 Mar 25;11(3):ENEURO.0259-23.2024. doi: 10.1523/ENEURO.0259-23.2024 (PMC10978822; doi:10.1523/ENEURO.0259-23.2024)

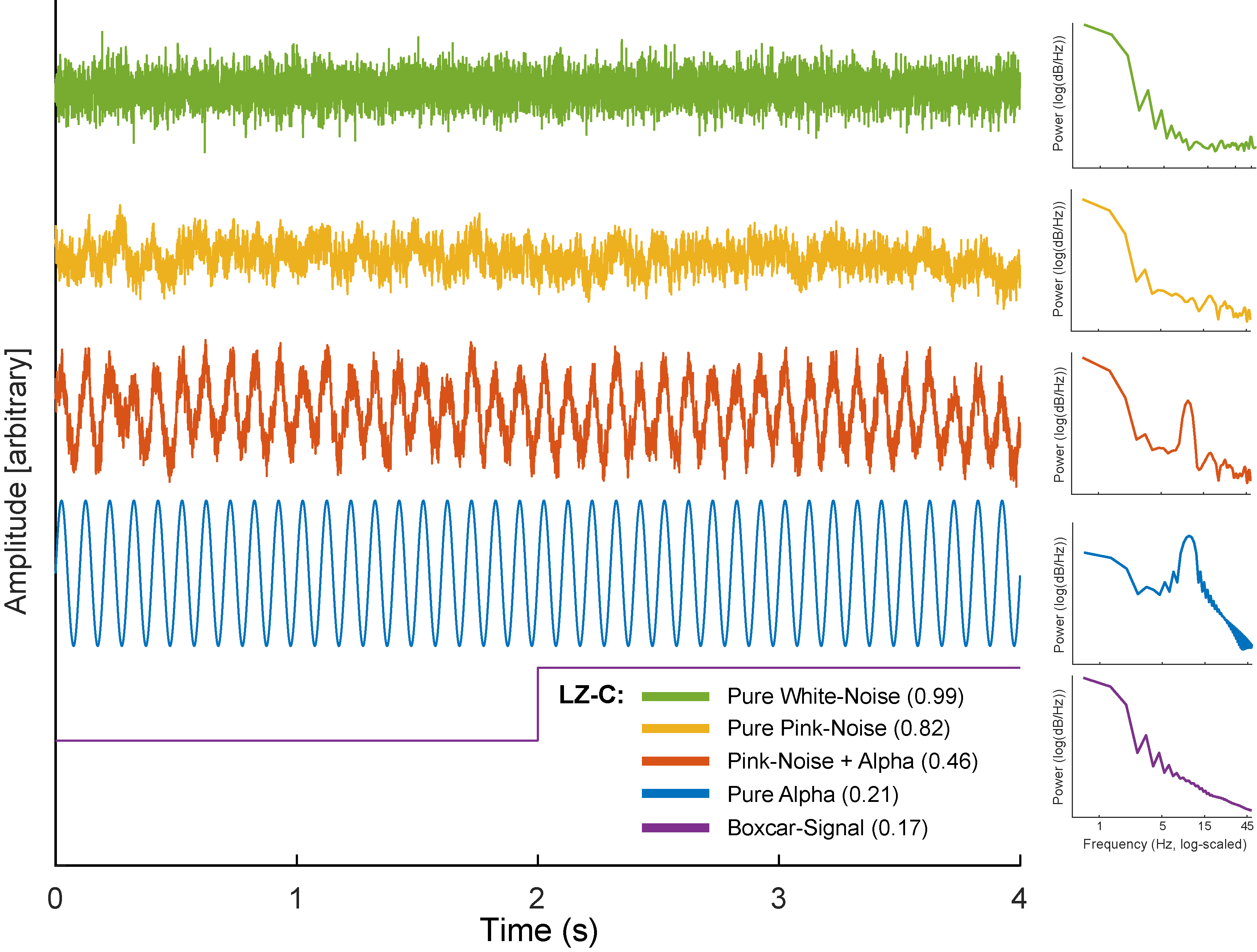

Supplement: Figure 1-4. — Illustration of the effect of signal regularity on resulting Lempel-Ziv complexity values and the shape of their power-spectra. The complexity values increase from a binary boxcar signal (purple) to a pure 10 Hz alpha oscillation (blue), further to the same oscillation with additional pink noise (red) and to pure pink noise (orange). Completely random white noise (green) has the highest complexity. Download Figure 1-4, TIF file. [file eneuro-11-ENEURO.0259-23.2024-s005.tif]

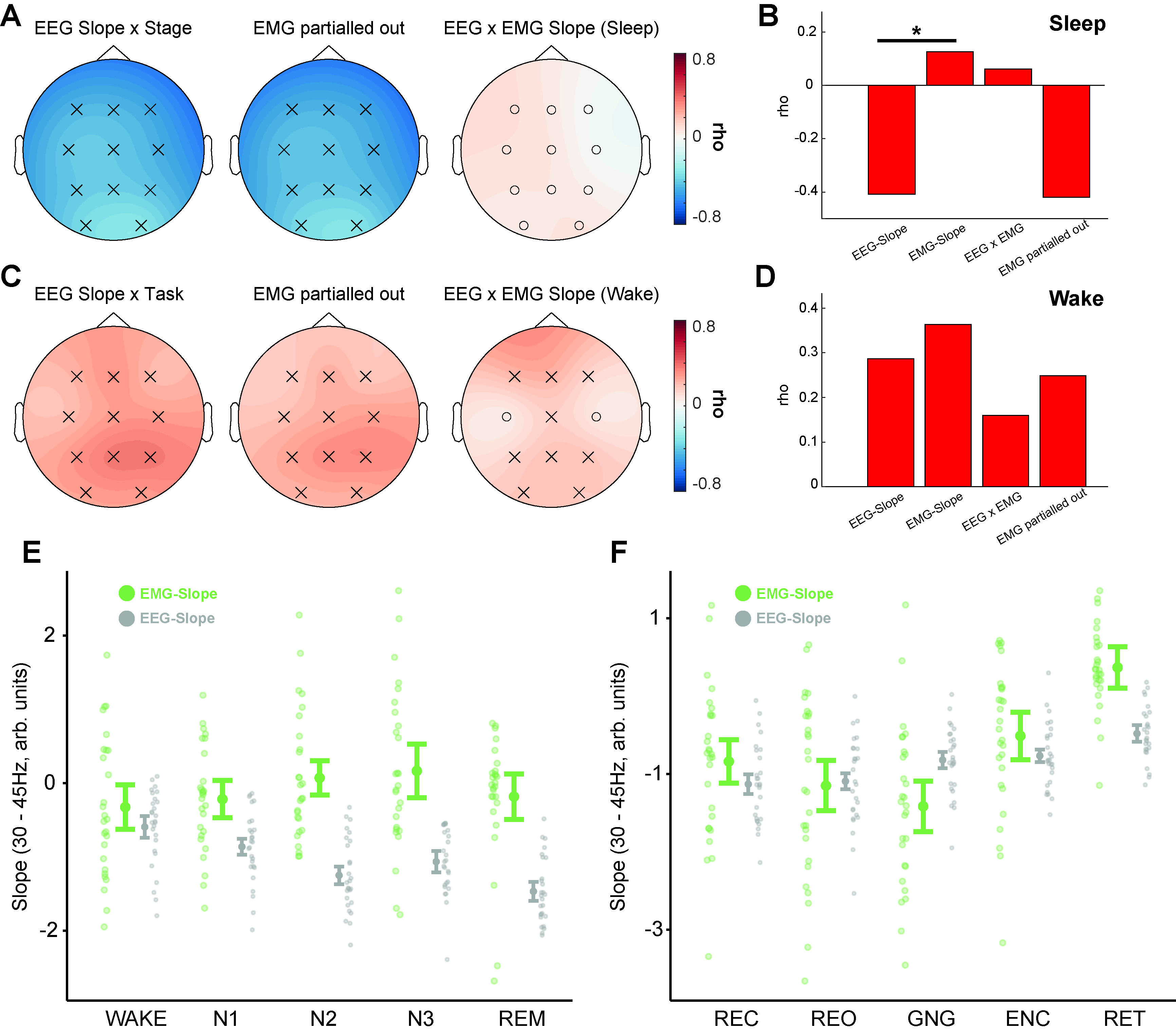

Supplement: Figure 2-1. — Control analyses including the narrowband spectral slope from the EMG. A: The negative correlations between EEG slope and sleep stage do not change when partialling out the EMG slope. B: While the average EEG slope is negatively correlated with sleep stage, the EMG slope is even slightly positively correlated with sleep stage and significantly different from the EEG slope correlation. C: The positive correlations between EEG slope and the cognitive tasks (ordered ascendingly regarding their slope) are not diminished when controlling for the EMG. D: While the correlation between the EMG slope and the tasks is slightly higher than between the EEG slope and the tasks, partialling out the EMG from the EEG slope does not significantly reduce the correlation. E & F: Differential modulation of the EEG & EMG slopes across sleep stages and tasks. Download Figure 2-1, TIF file. [file eneuro-11-ENEURO.0259-23.2024-s006.tif]

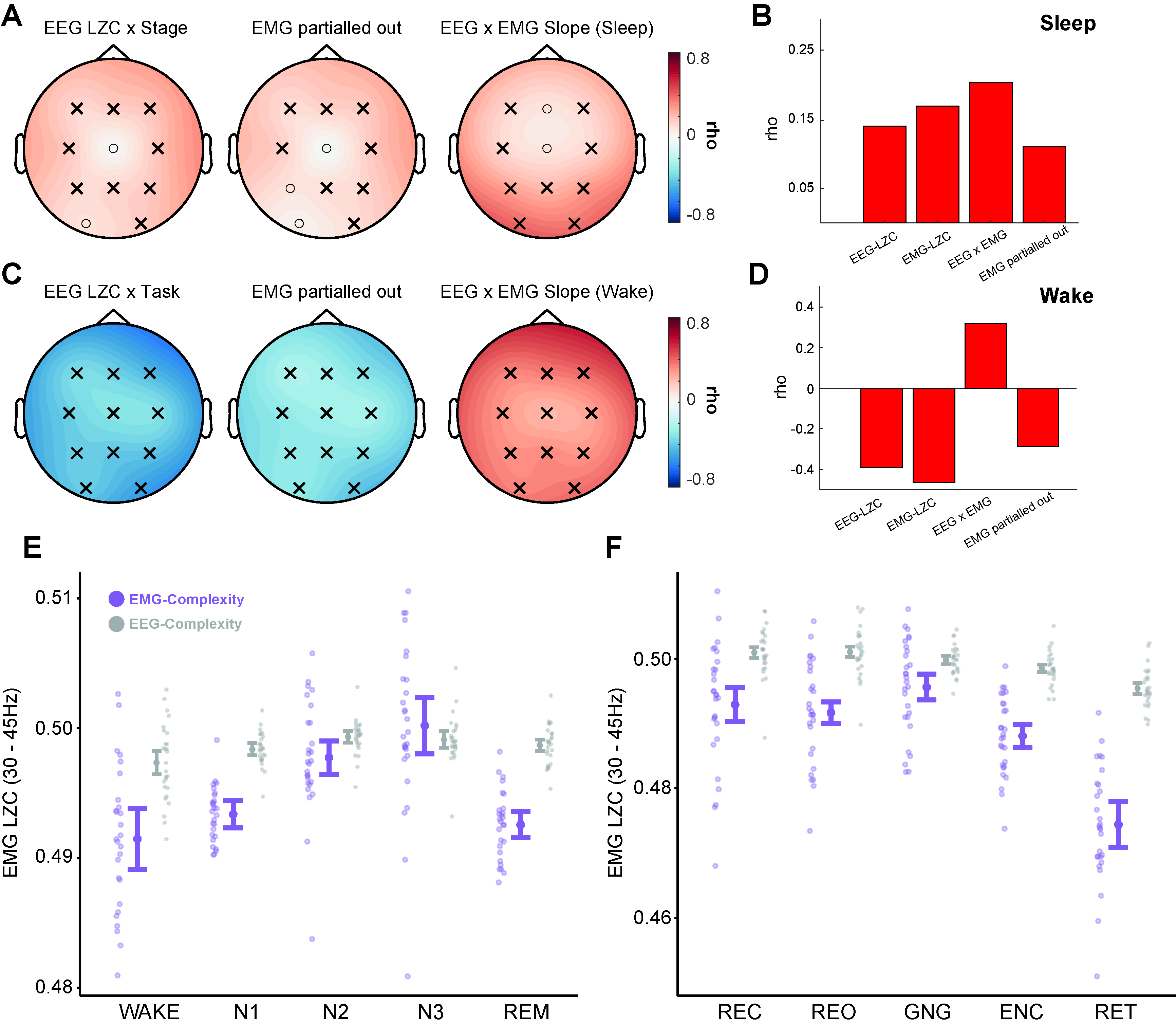

Supplement: Figure 2-2. — Control analyses including the narrowband Lempel-Ziv complexity (LZC) from the EMG. A: The positive correlations between EEG complexity and sleep stage do not change when partialling out the EMG complexity. B: While both, the average EEG and EMG complexity are positively correlated with sleep stage, the partial correlation controlling for EMG complexity does not shrink substantially. C: The negative correlations between EEG complexity and the cognitive tasks are not changed substantially by partialling out the EMG. D: Both, the average EEG and EMG complexity are negatively correlated with the tasks during wakefulness but the partial correlation between EEG complexity and the tasks controlled for the EMG is not significantly smaller. E & F: Differential modulation of the EEG & EMG complexity across sleep stages and tasks. Download Figure 2-2, TIF file. [file eneuro-11-ENEURO.0259-23.2024-s007.tif]

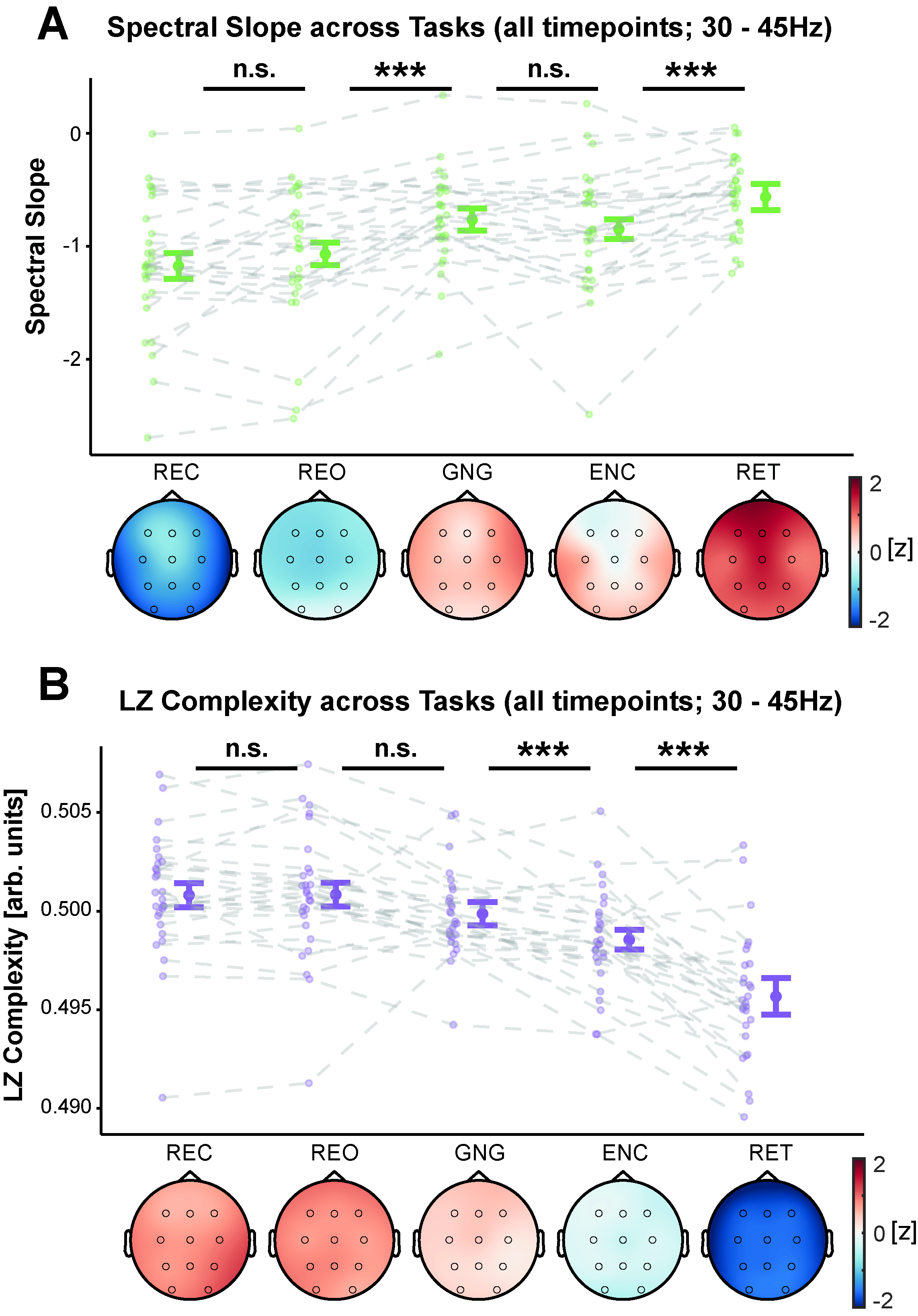

Supplement: Figure 4-1. — Slope and complexity (30 – 45Hz) across tasks averaged over all timepoints. Download Figure 4-1, TIF file. [file eneuro-11-ENEURO.0259-23.2024-s008.tif]

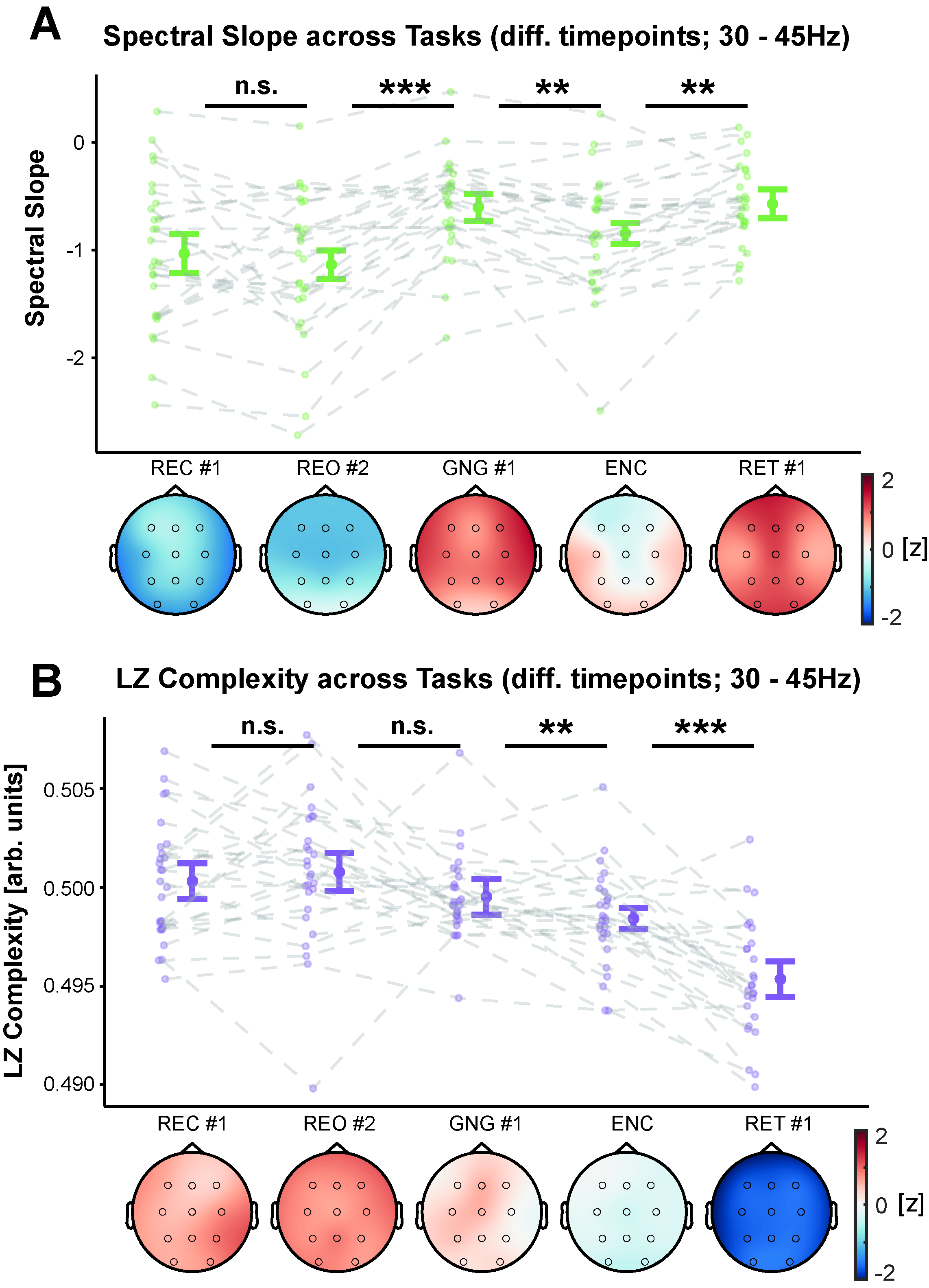

Supplement: Figure 4-2. — Slope and complexity (30 – 45Hz) across tasks using a different task-order (REC#1, GNG#1, ENC, REO#2, RET#1 instead of ENC, REC#2, REO#2, GNG#2, RET#1, cf., Figure 1). Download Figure 4-2, TIF file. [file eneuro-11-ENEURO.0259-23.2024-s009.tif]

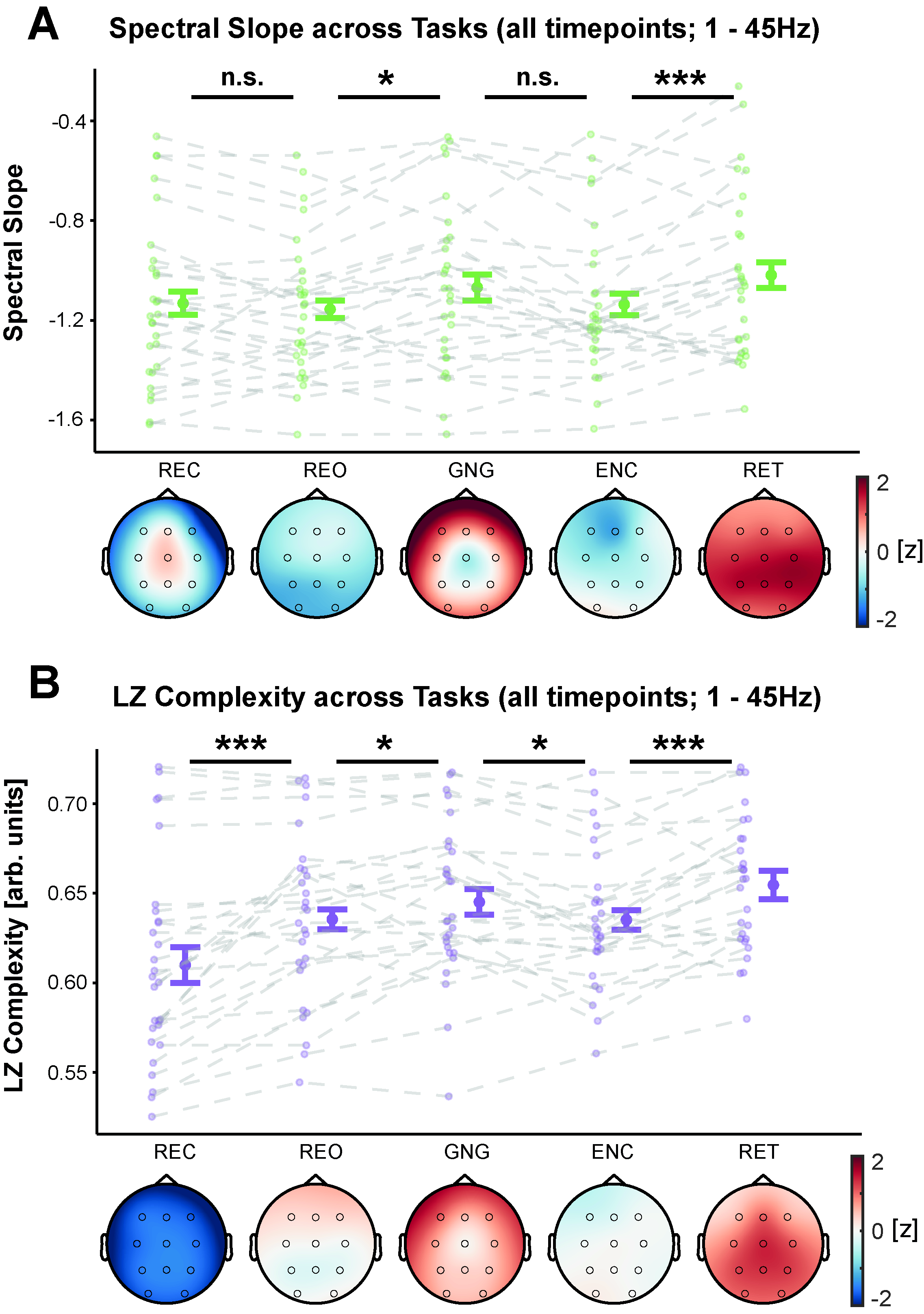

Supplement: Figure 5-1. — Slope and complexity (1 – 45Hz) across tasks averaged over all timepoints. Download Figure 5-1, TIF file. [file eneuro-11-ENEURO.0259-23.2024-s010.tif]

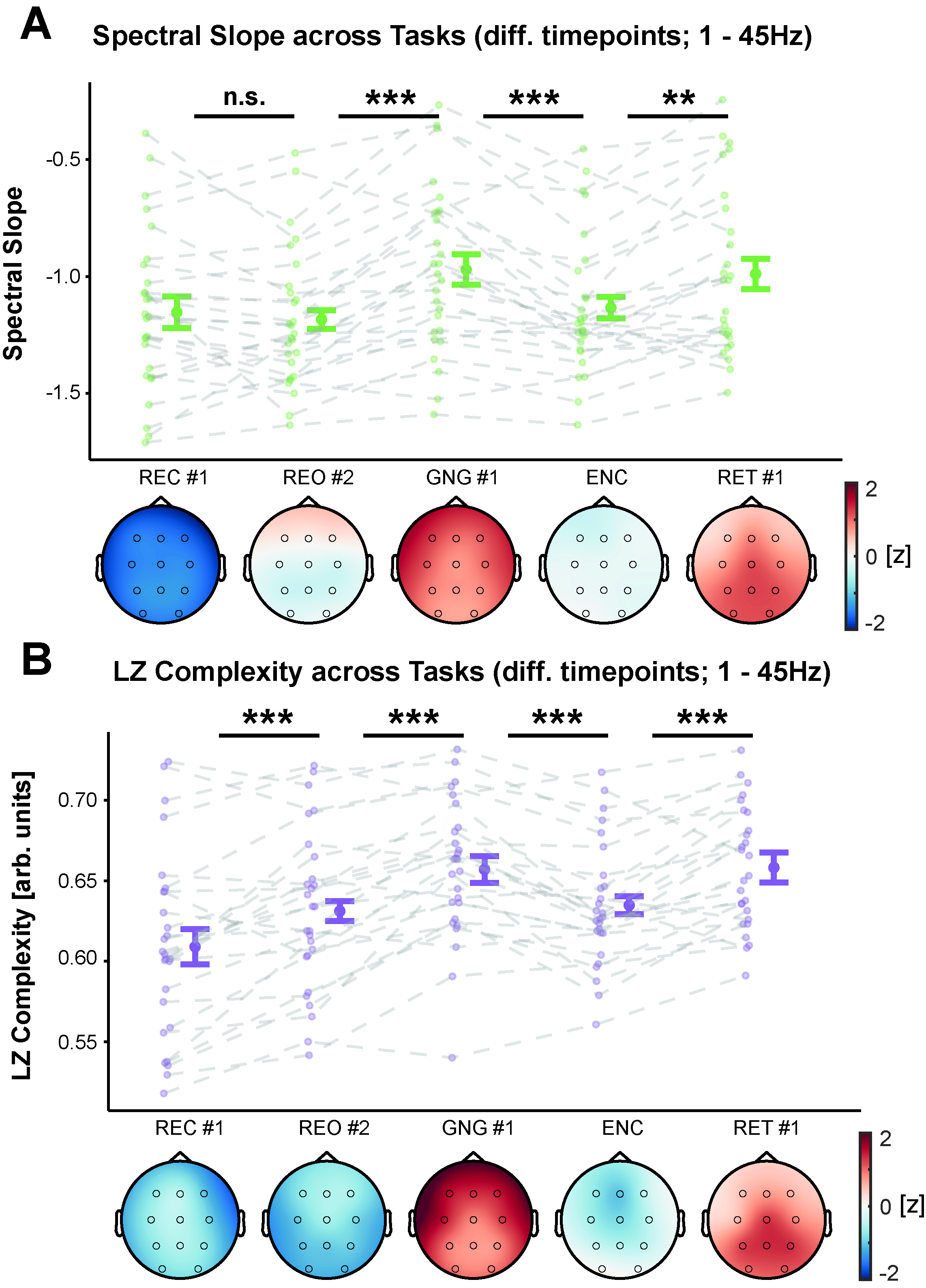

Supplement: Figure 5-2. — Slope and complexity (1 – 45Hz) across tasks using a different task-order (REC#1, GNG#1, ENC, REO#2, RET#1 instead of ENC, REC#2, REO#2, GNG#2, RET#1, cf., Figure 1). Download Figure 5-2, TIF file. [file eneuro-11-ENEURO.0259-23.2024-s011.tif]

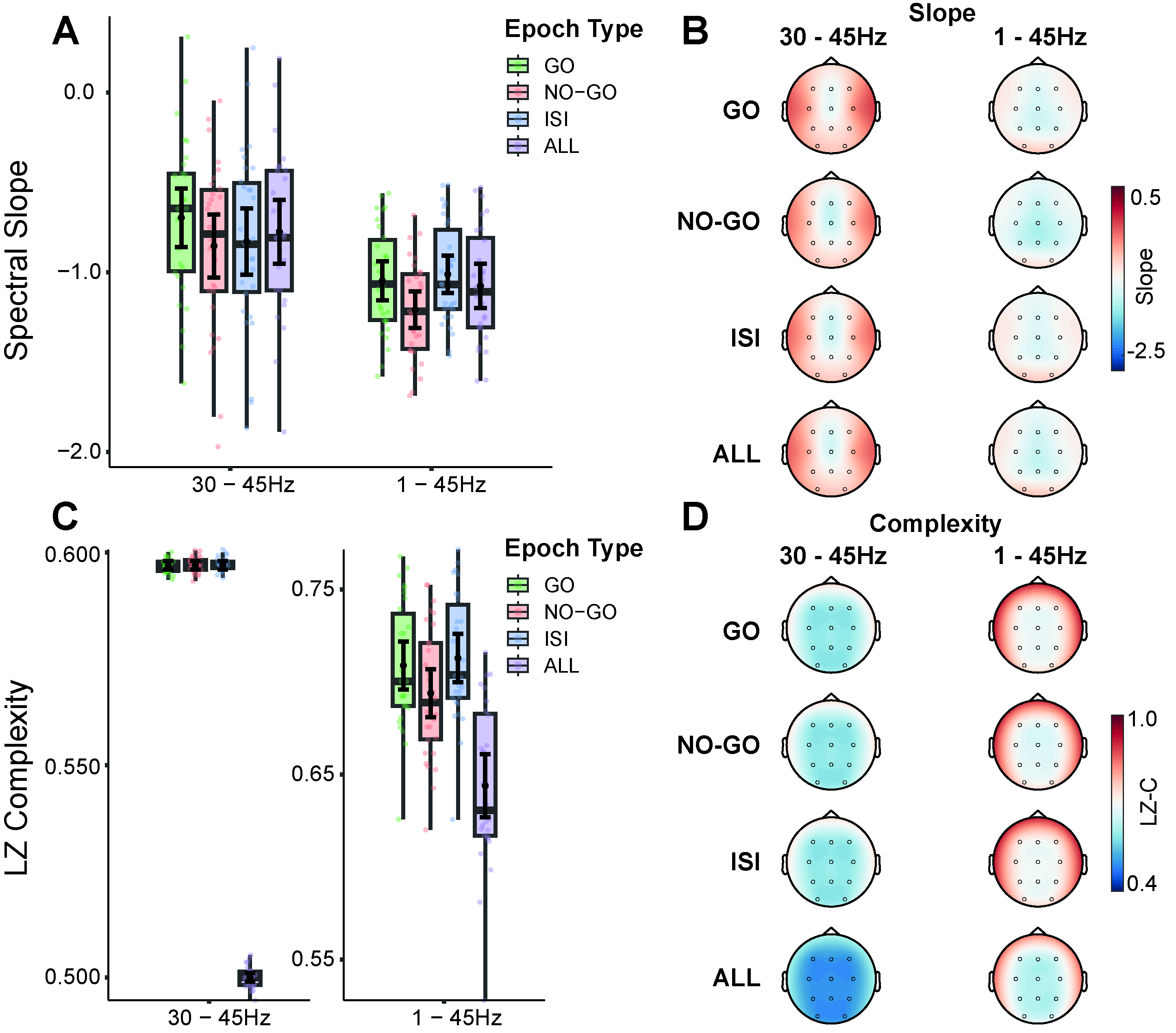

Supplement: Figure 5-3. — Effect of choosing 1s epochs around a stimulus (GO or NOGO) or 1s epochs during an interstimulus interval (ISI) or 4s epochs across the whole task. While the general epoch-length has a strong impact on the complexity estimates (C – D), neither the slope nor the complexity is strongly affected by different epoch-types (A – D). Download Figure 5-3, TIF file. [file eneuro-11-ENEURO.0259-23.2024-s012.tif]

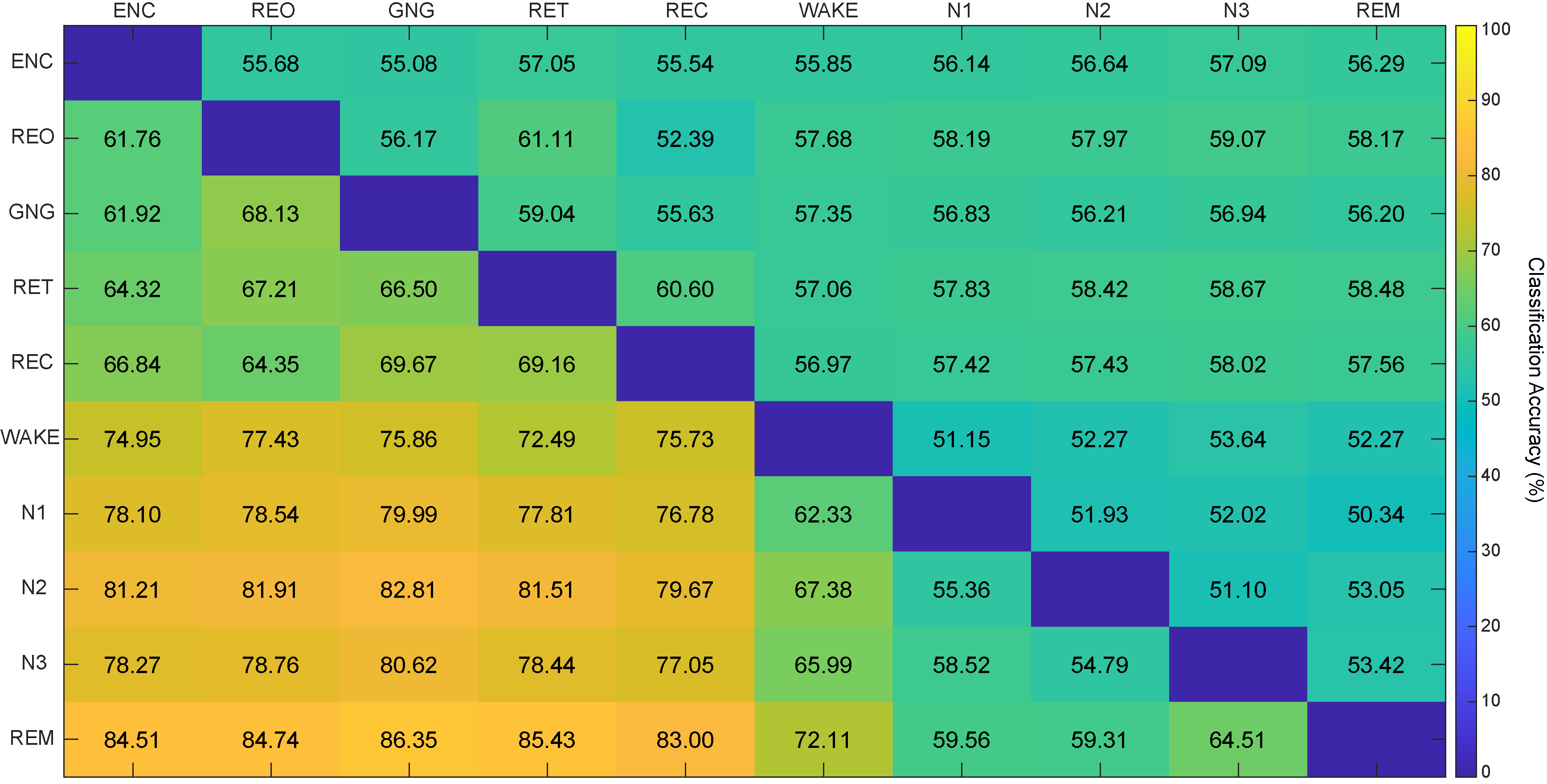

Supplement: Figure 6-1. — Classification accuracy for all pairwise combinations of sleep stage and task (30 – 45Hz). Upper triangular matrix shows the results for Lempel-Ziv complexity and lower triangular matrix for the spectral slope. Data was pooled over all lab-visits for each subject. Download Figure 6-1, TIF file. [file eneuro-11-ENEURO.0259-23.2024-s013.tif]

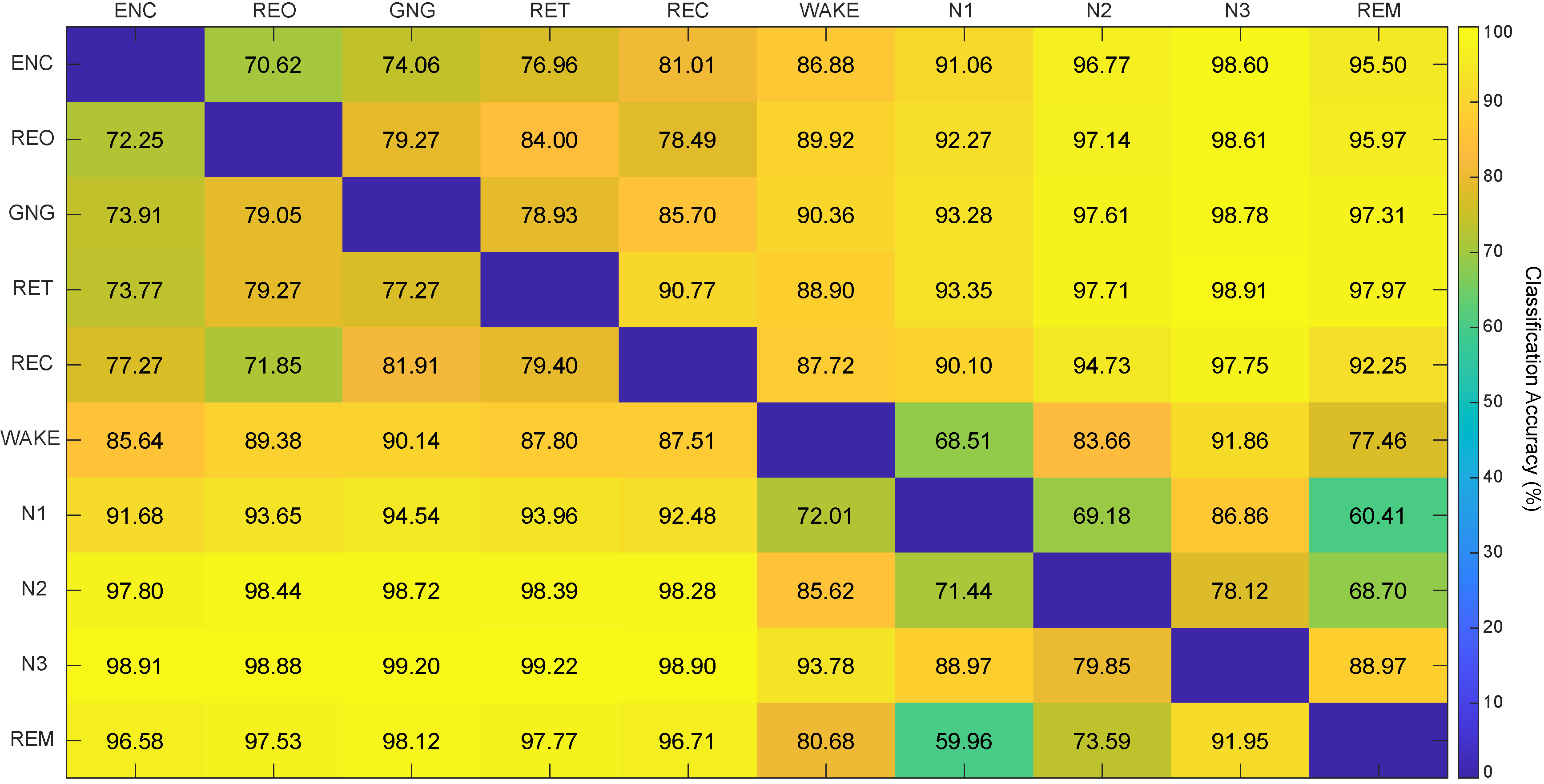

Supplement: Figure 6-2. — Classification accuracy for all pairwise combinations of sleep stage and task (1 – 45Hz). Upper triangular matrix shows the results for Lempel-Ziv complexity and the lower triangular matrix for the spectral slope. The data was pooled over all lab-visits for each subject. Download Figure 6-2, TIF file. [file eneuro-11-ENEURO.0259-23.2024-s014.tif]

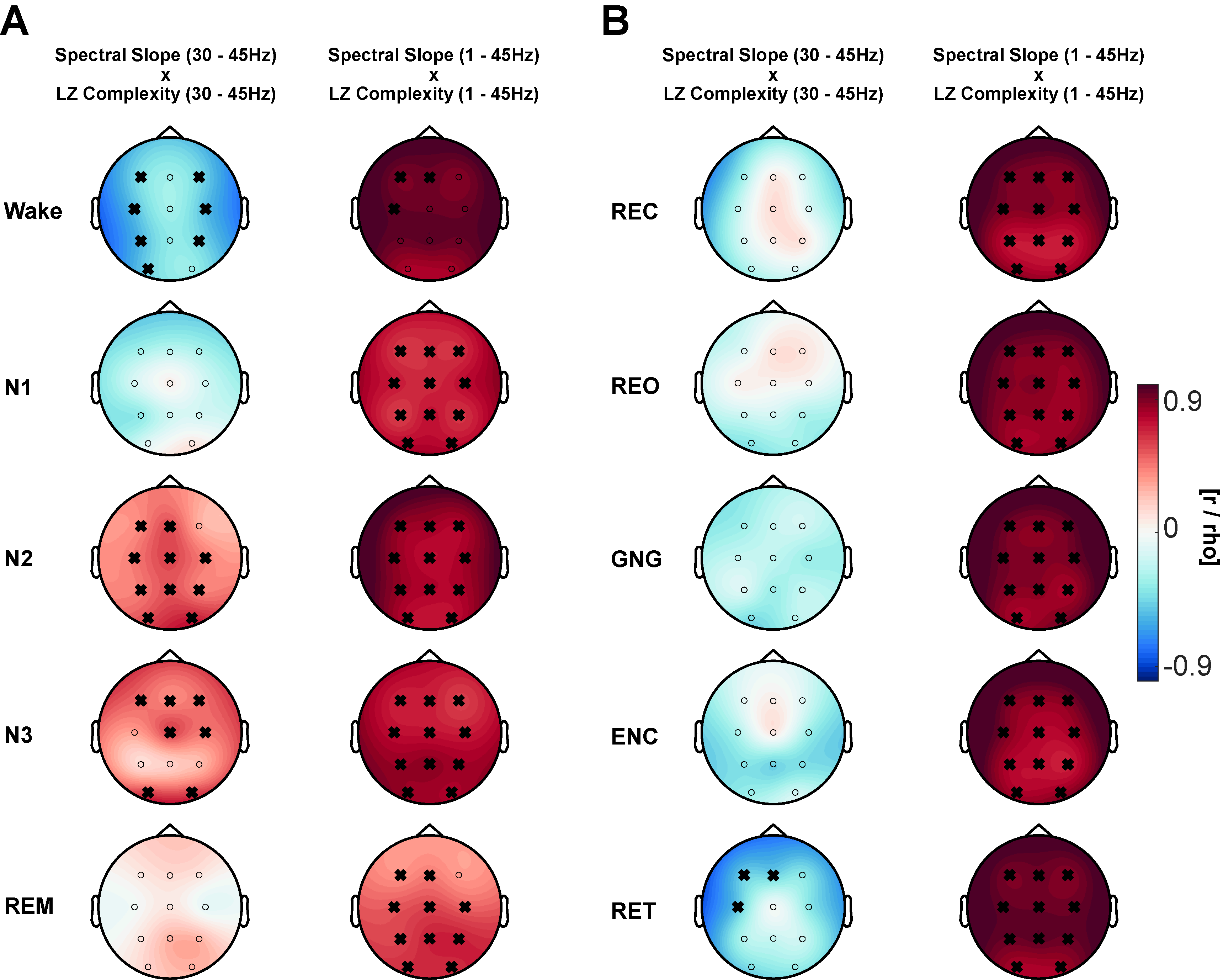

Supplement: Figure 6-3. — Correlations between spectral slope and Lempel-Ziv (LZ) complexity from 30 – 45Hz and 1 – 45Hz. The sleep (A) and task (B) data per subject were averaged across all lab-sessions. For task data, only the evening assessments highlighted by the dashed dark-green rectangle in Figure 1 were considered. Significant correlations (p ≤ .050 after correcting for false discovery rate) are highlighted with a cross on the topographical maps (color codes for the size and directionality of the correlation coefficients). Download Figure 6-3, TIF file. [file eneuro-11-ENEURO.0259-23.2024-s015.tif]

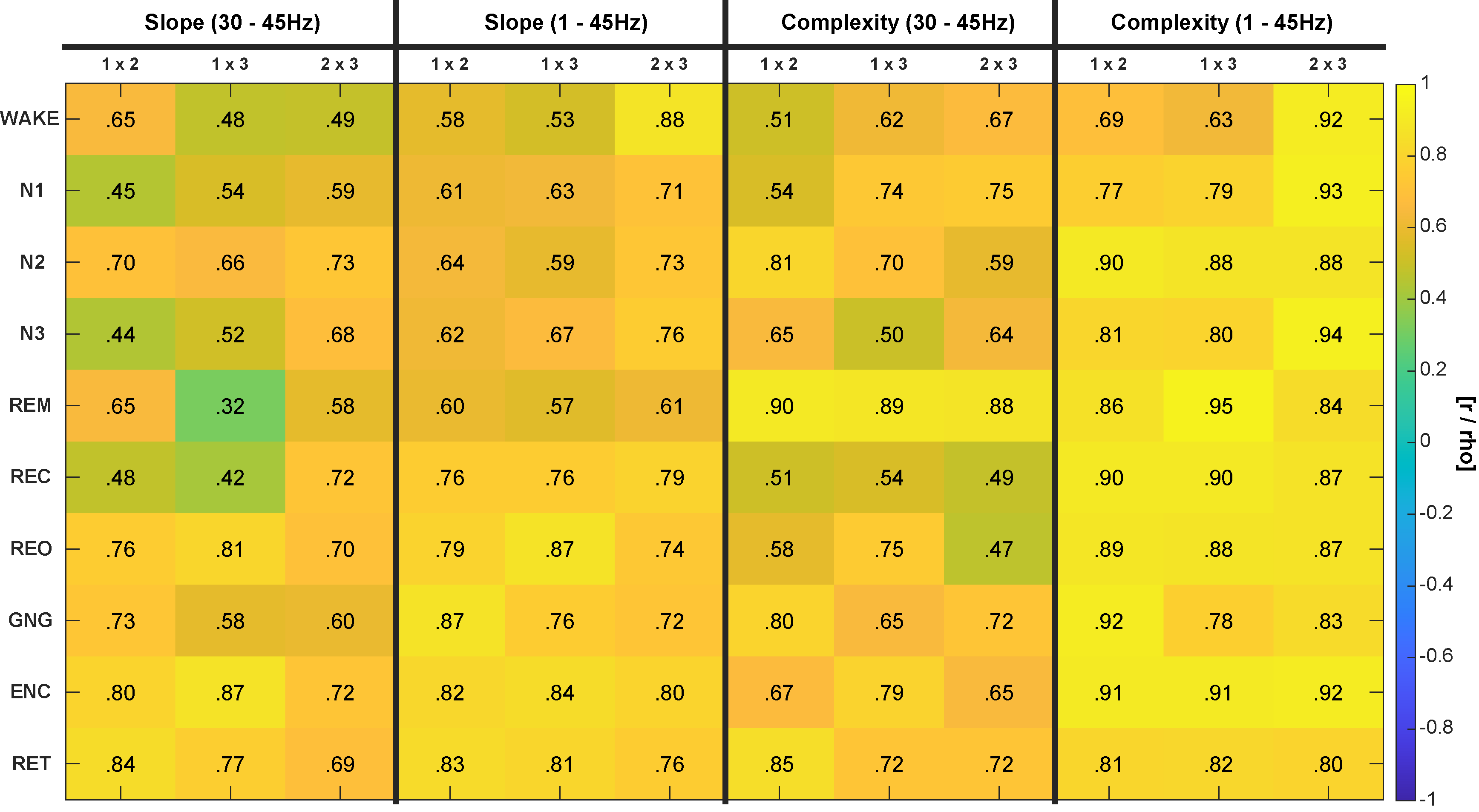

Supplement: Figure 6-4. — Robustness of the spectral slope and Lempel-Ziv complexity across lab-visits. Correlation coefficients over all electrodes for each parameter between the three experimental recordings (1 x 2, 1 x 3 and 2 x 3). Each of the experimental recordings refers to one lab-visit per subject. Download Figure 6-4, TIF file. [file eneuro-11-ENEURO.0259-23.2024-s016.tif]

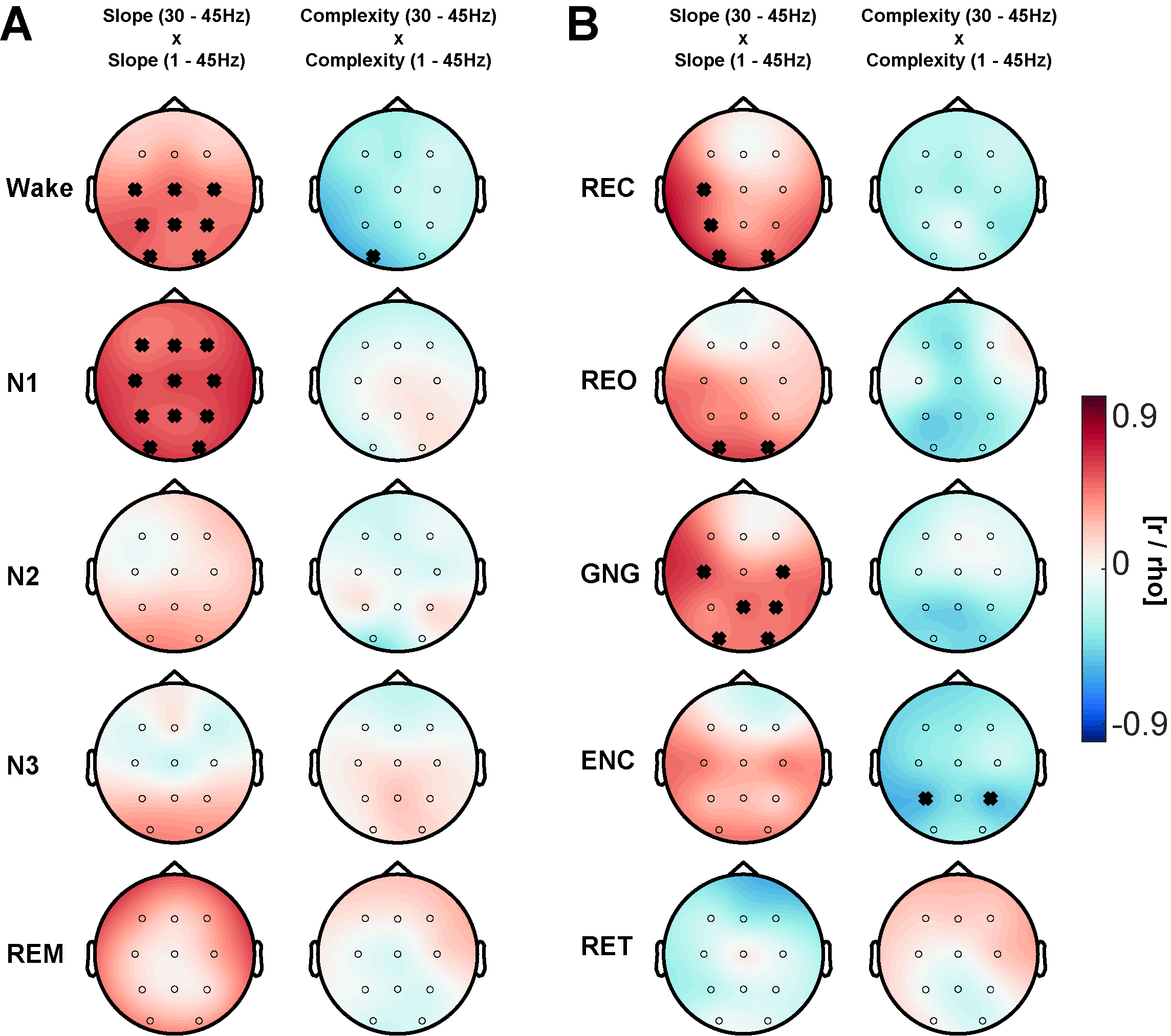

Supplement: Figure 6-5. — Correlation of the slope and complexity with themselves in the narrow- or broadband frequency range during sleep (A) and wakefulness (B). Download Figure 6-5, TIF file. [file eneuro-11-ENEURO.0259-23.2024-s017.tif]

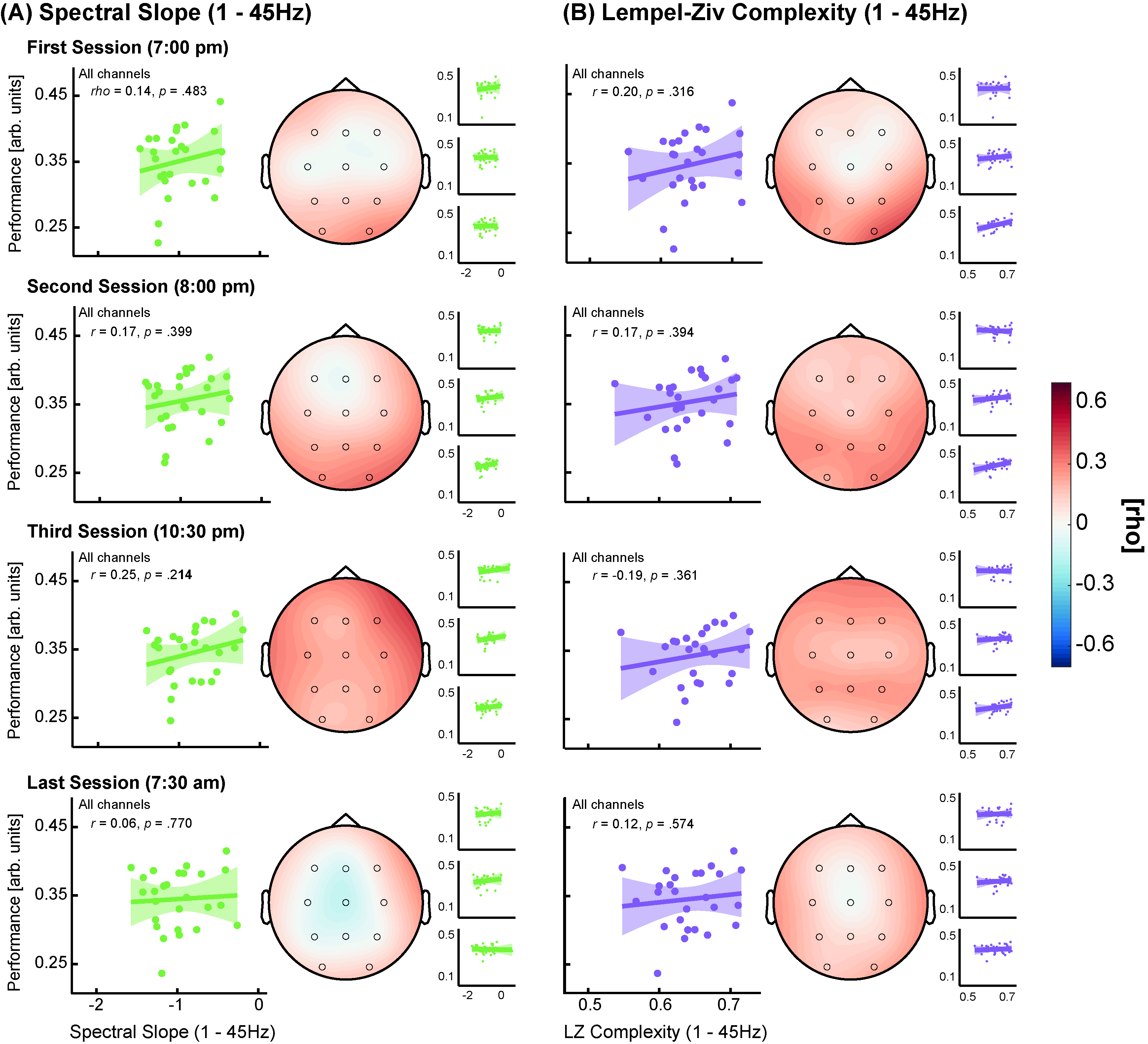

Supplement: Figure 7-1. — Results when using the broadband (1 – 45Hz) frequency range. No significant relationships emerged for the spectral slope and Lempel-Ziv complexity, even though correlations were consistently positive for both parameters. Download Figure 7-1, TIF file. [file eneuro-11-ENEURO.0259-23.2024-s018.tif]

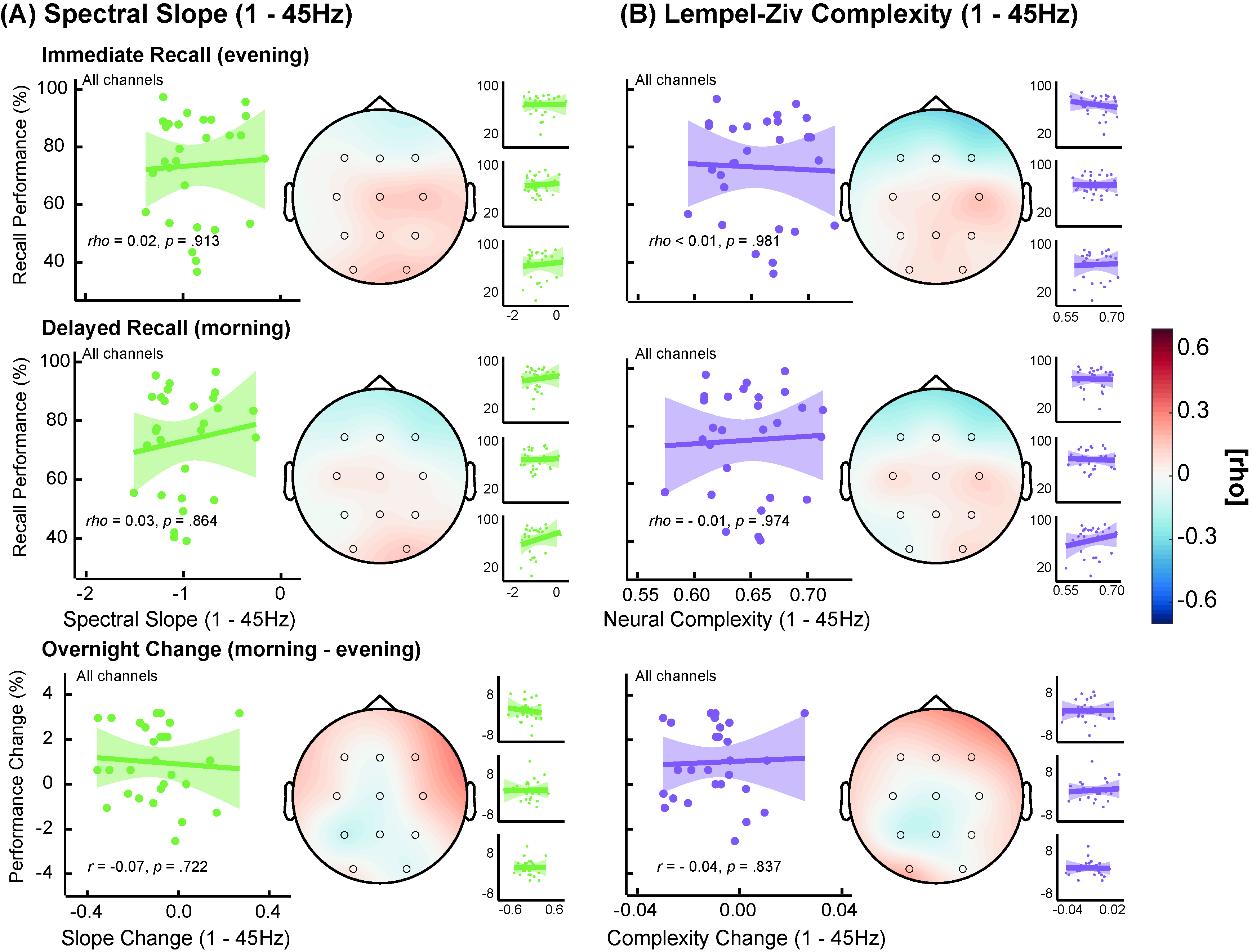

Supplement: Figure 8-1. — Results when using the broadband 1 – 45Hz frequency range. No relationship observable between recall performance and slope or complexity. Download Figure 8-1, TIF file. [file eneuro-11-ENEURO.0259-23.2024-s019.tif]
